# Estimating the minimal important change of single-item measures using the adjusted predictive modeling method or the longitudinal confirmatory factor analysis method

**DOI:** 10.1007/s11136-025-04134-3

**Published:** 2026-01-09

**Authors:** Berend Terluin, Yong Hao Pua, Piper Fromy, Andrew Trigg, Babette van der Zwaard, Jakob B. Bjorner

**Affiliations:** 1 Department of General Practice, Amsterdam UMC, Vrije Universiteit Amsterdam, De Boelelaan 1117, Amsterdam, 1081 HV The Netherlands; 2 Amsterdam Public Health Research Institute, Amsterdam, The Netherlands; 3 Department of Physiotherapy, Singapore General Hospital, Singapore, Singapore; 4 Medicine Academic Programme, Duke-NUS Graduate Medical School, Singapore, Singapore; 5 SeeingTheta, 2 Chemin des Vaux, 49400 Saumur, France; 6 Clinical Statistics and Analytics, Bayer plc, Reading, UK; 7 Department of Orthopedic Surgery, Jeroen Bosch Hospital, ‘s-Hertogenbosch, The Netherlands; 8 QualityMetric and IQVIA Company, Johnston, RI USA; 9 Department of Public Health, University of Copenhagen, Copenhagen, Denmark; 10 National Research Centre for the Working Environment, Copenhagen, Denmark

**Keywords:** Minimal important change, Meaningful change threshold, Single item measure, Transition ratings, Patient-reported outcome measure, Adjusted predictive modeling method, Longitudinal confirmatory factor analysis method

## Abstract

**Purpose:**

Recently developed minimal important change (MIC) estimation methods recover the mean individual MIC in a sample. These methods are the adjusted predictive modeling (APM) method and the longitudinal confirmatory factor analysis (LCFA) method. Both methods require LCFA of patient-reported outcome measure (PROM) data. In the APM-method, LCFA is used to estimate the reliability of the transition ratings, whereas in the LCFA-method, LCFA is used to estimate the latent MIC. However, LCFA cannot be performed if the PROM is a single item measure (SIM). Adding an auxiliary variable, that is correlated with the PROM, to the LCFA-model may be a solution. We developed three different LCFA-models in which an auxiliary variable is included. In this simulation study, we assessed the performance of the APM- and LCFA-methods to recover the true MIC of an SIM. We applied both methods to a real dataset in which the SIM was a numeric rating scale for pain.

**Methods:**

We simulated 15,552 samples, varying in 11 parameters, and estimated the APM-based and LCFA-based MICs.

**Results:**

The APM-method performed well, except if the proportion improved was high or low, and the present state bias (PSB) was high. The LCFA-method performed well, irrespective of the proportion improved and the PSB. In the real data, the LCFA-based MIC was 17 (on a 100-point scale), whereas the estimated APM-based MIC was 4 points higher, probably due to a high proportion improved and a high PSB.

**Conclusion:**

The MIC of an SIM can be accurately estimated using an auxiliary PROM.

**Supplementary Information:**

The online version contains supplementary material available at 10.1007/s11136-025-04134-3.

## Introduction

The minimal important change (MIC) of a patient-reported outcome measure (PROM) represents the PROM change score that corresponds to the smallest within-individual change^1^ in the construct that the PROM is measuring, that the average patient values as important [1]. Anchor-based MIC methods employ an “anchor” that conveys the patients’ perspective on the change they have experienced. A frequently used anchor is the patient-reported transition (or global impression of change) rating. A typical example is: “Please choose the response that best describes the overall change in your < symptom/overall status/etc.> since you started taking the study medication. Much worse, A little worse, No change, A little better, Much better” [2]. In this case the threshold between “No change” and “A little better” might be thought of as indicative of the MIC. The MIC can be conceptualized as the mean of individual MICs [3–5]. Simulation studies have shown that this MIC can be accurately estimated using one of the following methods: (1) the (improved) adjusted predictive modeling (APM) method [6], the longitudinal item response theory (LIRT) method [7], and the longitudinal confirmatory factor analysis (LCFA) method [8]. Because the LCFA-method is statistically equivalent to the LIRT-method, we will focus on just two methods, the APM-method and the LCFA-method.

The APM- and LCFA-methods are quite different. The LCFA-method is rooted in latent variable modeling, and directly estimates the latent MIC (i.e., the MIC in the metric of the latent construct underlying the data). The latent MIC is then translated into the MIC in the metric of the PROM [8]. The APM-method is rooted in logistic regression analysis, and calculates the PROM change score that is equally likely to occur in the improved and not-improved groups (the so-called predictive modeling MIC) [9]. This MIC is subsequently adjusted for the bias caused by the proportion improved [5, 6]. One of the parameters that needs to be taken into account in the adjustment, is the reliability of the transition ratings (TRs), which must be estimated using LCFA [10]. So, both MIC estimation methods require the application of LCFA, but for different purposes. In the LCFA-method, LCFA is the main analysis, used to estimate the latent MIC, whereas, in the APM-method, logistic regression is the main analysis, and LCFA is only used to estimate the TRs reliability.

**Fig. 1:**
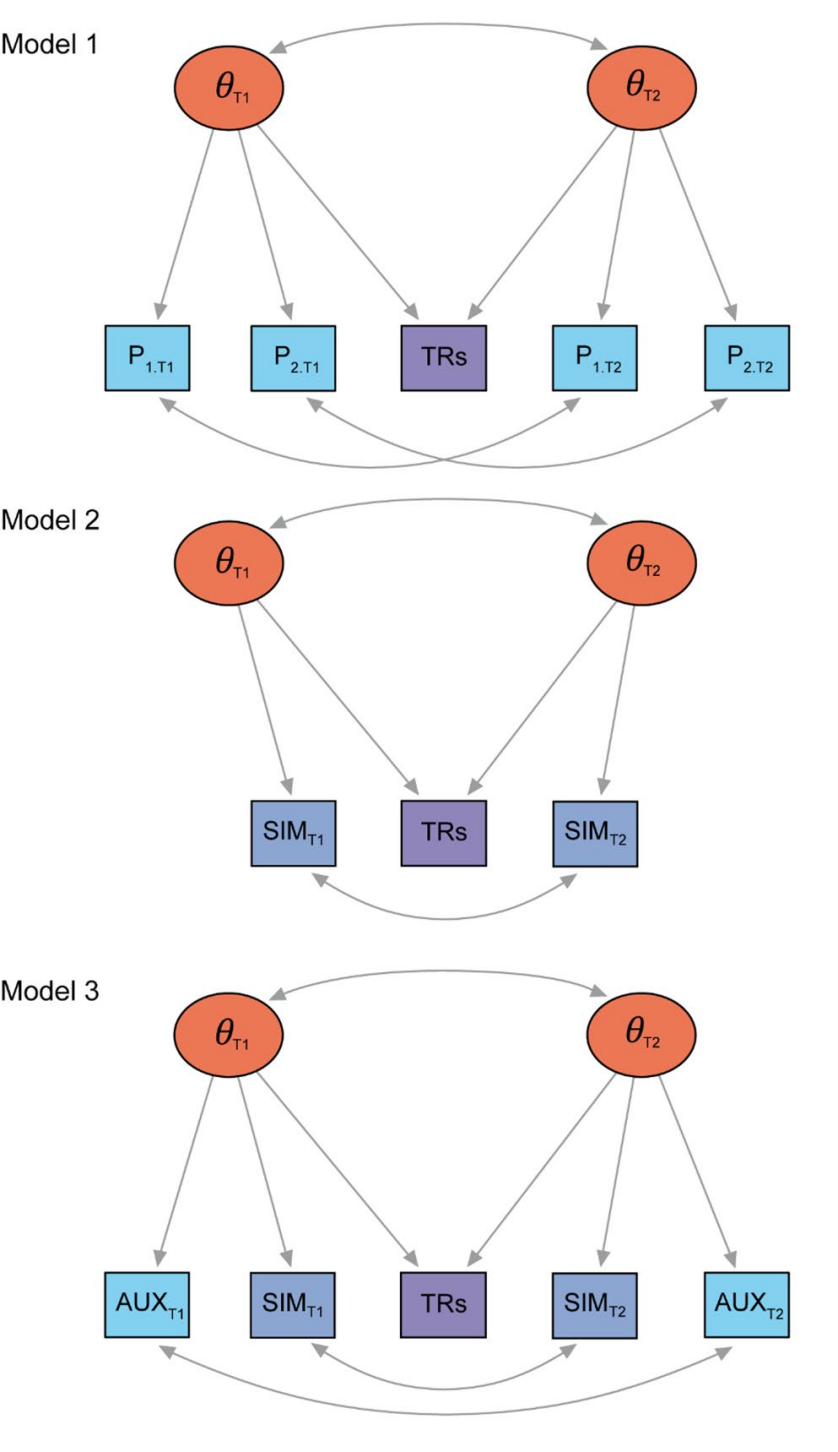
Three longitudinal confirmatory factor analysis (LCFA) models. Ovals represent latent constructs (factors, $\:\theta\:$) at baseline (T1) and follow-up (T2). Rectangles represent manifest variables. P indicates PROM items (indexed by the number of the item and the time). SIM indicates single-item measures (indexed by the time). TRs indicates a transition ratings item. AUX represents auxiliary variables (indexed by the time). Single-headed arrows represent factor loadings, and double-headed arrows represent correlations. Model 1 is a model with a two-item PROM. Each factor is defined by the minimally required three indicators. Model 2 is a model with an SIM. This model is not estimable. Model 3 is a model with an SIM, enriched with an auxiliary variable. This model is again estimable

The APM- and LCFA-methods work well with a multi-item PROM, but they cannot handle a single-item measure (SIM), such as a numeric rating scale (NRS), a visual analogue scale (VAS), a performance measure, or a digital health technology measure. The reason for this is that an LCFA requires that each latent factor has at least three indicators (items) to be identified. Figure 1, model 1, shows a typical LCFA-model fitted to a PROM with two items, measured at two time points, T1 and T2, and a TRs item. The latent factor at T1 ($\:{\theta\:}_{\mathrm{T}1}$) has three indicators, two PROM items (P_1.T1_ and P_2.T1_) and the TRs item, whereas the latent factor at T2 ($\:{\theta\:}_{\mathrm{T}2}$) has also three indicators, two PROM items (P_1.T2_ and P_2.T2_) and the TRs item. Note that the TRs item serves as an indicator of both latent factors.

A similar model for an SIM is shown in Fig. 1, model 2. This model cannot be estimated because two indicators for each latent factor provide too little information to identify the factor. A possible solution to fit an LCFA to SIMs, is to “enrich” the model with another (auxiliary) variable (AUX), as shown in Fig. 1, model 3. In many studies in which SIMs are used, one or more other PROMs are administered simultaneously. These other PROMs usually do not target the exact same construct as the SIM, but they do often correlate with the SIM, indicating some overlap of the constructs measured by the SIM and the AUX. With the help from an AUX, each latent factor has again three indicators (Fig. 1, model 3), and the LCFA-model can be estimated.

The question is whether such an AUX-enriched model can be used to estimate the MIC of an SIM using either the APM- or the LCFA-method. We explored this through the simulation of multiple datasets with SIMs, TRs, and AUXs, and tested three different ways to enrich LCFA-models with an AUX. Additionally, for illustration, we analyzed a real dataset.

## Methods

### Data simulation

Suppose an SIM targets a construct of interest, and an AUX targets a different, but correlated, construct. As an example, we can think of an SIM for “physical functioning” and an AUX for “activities of daily living”. Given that a particular construct can be thought of as consisting of several components, the correlation between an SIM and an AUX implies that both measures target one or more components that are shared by both constructs. Therefore, an AUX can target one or more construct components that are also targeted by an SIM *and* additionally target other construct components that are not targeted by the SIM, and vice versa. In other words, an AUX can target “more” or “less” than the construct that is targeted by the SIM. We used this principle to generate SIM and AUX item responses based on four independent latent constructs, labeled $\:{\theta\:}^{\mathrm{A}}$, $\:{\theta\:}^{\mathrm{B}}$, $\:\xi\:$ and $\:\eta\:$, underlying the SIM, the AUX, and the TRs. Specifically, the construct of interest ($\:\theta\:$) consisted of two components ($\:{\theta\:}^{\mathrm{A}}$ and $\:{\theta\:}^{\mathrm{B}}$). $\:{\theta\:}^{\mathrm{A}}$, $\:{\theta\:}^{\mathrm{B}}$, and $\:\eta\:$ were underlying the SIM scores, meaning that the SIM measured the construct of interest ($\:\theta\:$) and something else ($\:\eta\:$) that was unrelated to $\:\theta\:$. Furthermore, the AUX was simulated as a multi-item PROM with $\:{\theta\:}^{\mathrm{A}}$ and $\:\xi\:$ as underlying constructs. Therefore, the SIM and the AUX shared $\:{\theta\:}^{\mathrm{A}}$ as a common underlying construct. So, the AUX could target both more ($\:\xi\:$) and/or less ($\:{\theta\:}^{\mathrm{B}}$) than was targeted by the SIM, depending on the strengths of $\:\xi\:$ and/or $\:{\theta\:}^{\mathrm{B}}$. Details of the simulation process are described in Online Resource, Sect. 1.

The data were simulated at two time points (T1 and T2), varying the change in $\:\theta\:$ to create various proportions improved, relative to a simulated true MIC of 0.8 in the metric of the change in $\:\theta\:$.^2^ TRs were simulated based on a weighted change of $\:\theta\:$ between T1 and T2, reflecting varying degrees of present state bias (PSB) [11]. Moreover, we varied the strengths of $\:{\theta\:}^{\mathrm{B}}$ and $\:\xi\:$, relative to $\:{\theta\:}^{\mathrm{A}}$.

We varied 11 parameters across the simulated datasets (Table 1). There were 15,552 different combinations of parameters; for each combination we simulated one dataset (with *n* = 2000). The R-code used for the data simulation and MIC estimation is provided in the Online Resource, Sect. 7. We used the R-package “mirt”, version 1.41 [12] within the statistical program R, version 4.3.2 [13] to simulate AUX item response data.

**Table 1:** Parameters varying across the simulated datasets

Parameter	Values	Explanation
Mean latent trait$\:\theta\:$at T1 ($\:\overline{{\theta\:}_{\mathrm{T}1}}$)	− 1, 0, 1	$\:\overline{{\theta\:}_{\mathrm{T}1}}$reflects the average (severity) level of the latent trait at T1
SD latent change (SD($\:\varDelta\:\theta\:$))	0.85, 1.15	SD ($\:\varDelta\:\theta\:$) reflects the variability in the latent change ($\:\varDelta\:\theta\:$) between T1 and T2
Correlation between$\:{\theta\:}_{\mathrm{T}1}$and$\:\varDelta\:\theta\:$	− 0.50, 0.00	Values are correlation coefficients
Reliability of SIM_T1_	0.50, 0.80	Values are reliability coefficients
Reliability of the TRs	0.40, 0.70	Values are reliability coefficients
Present state bias (PSB)	0, 0.4, 0.8	Proportion of$\:{\theta\:}_{\mathrm{T}2}-\overline{{\theta\:}_{\mathrm{T}1}}$that is included in the weighted latent change ($\:{\varDelta\:\theta\:}_{w}$)
Proportion improved	0.2, 0.5, 0.8	Proportion of patients having a true latent change ($\:\varDelta\:\theta\:$) greater than their individual MIC
Relative strength of the unique AUX factor ($\:\xi\:$)	0, 0.2, 0.5	Values are ratios of the mean slope parameter of$\:\xi\:$to the mean slope parameter of$\:{\theta\:}^{\mathrm{A}}$
Relative strength of$\:{\theta\:}^{\mathrm{B}}$	0, 0.5, 1	Ratio of the SD of$\:{\theta\:}^{\mathrm{B}}$to the SD of$\:{\theta\:}^{\mathrm{A}}$
Correlation between$\:{\xi\:}_{\mathrm{T}1}$and$\:{\xi\:}_{\mathrm{T}2}$	0.50, 0.90	Values are correlation coefficients
Correlation between$\:{\eta\:}_{\mathrm{T}1}$and$\:{\eta\:}_{\mathrm{T}2}$	0.50, 0.90	Values are correlation coefficients

SD, Standard deviation; T1, Baseline; T2, Follow-up; SIM, single-item measure; TRs, transition ratings; AUX, auxiliary patient-reported outcome measure

### Analysis

#### LCFA-models

The APM-method requires estimation of the reliability of the TRs through LCFA [6]. For this purpose, the LCFA-model only requires that same indicators (items) are allowed to correlate over time [10]. The LCFA-method requires estimation of the latent MIC through LCFA [8]. For this purpose, the LCFA-model needs to be specified as a measurement invariance model in order to correctly estimate the latent change metric. Irrespective of the model specification, the AUX variable can be included in an LCFA-model as a single item or as a sum score of multiple items.

**Fig. 2:**
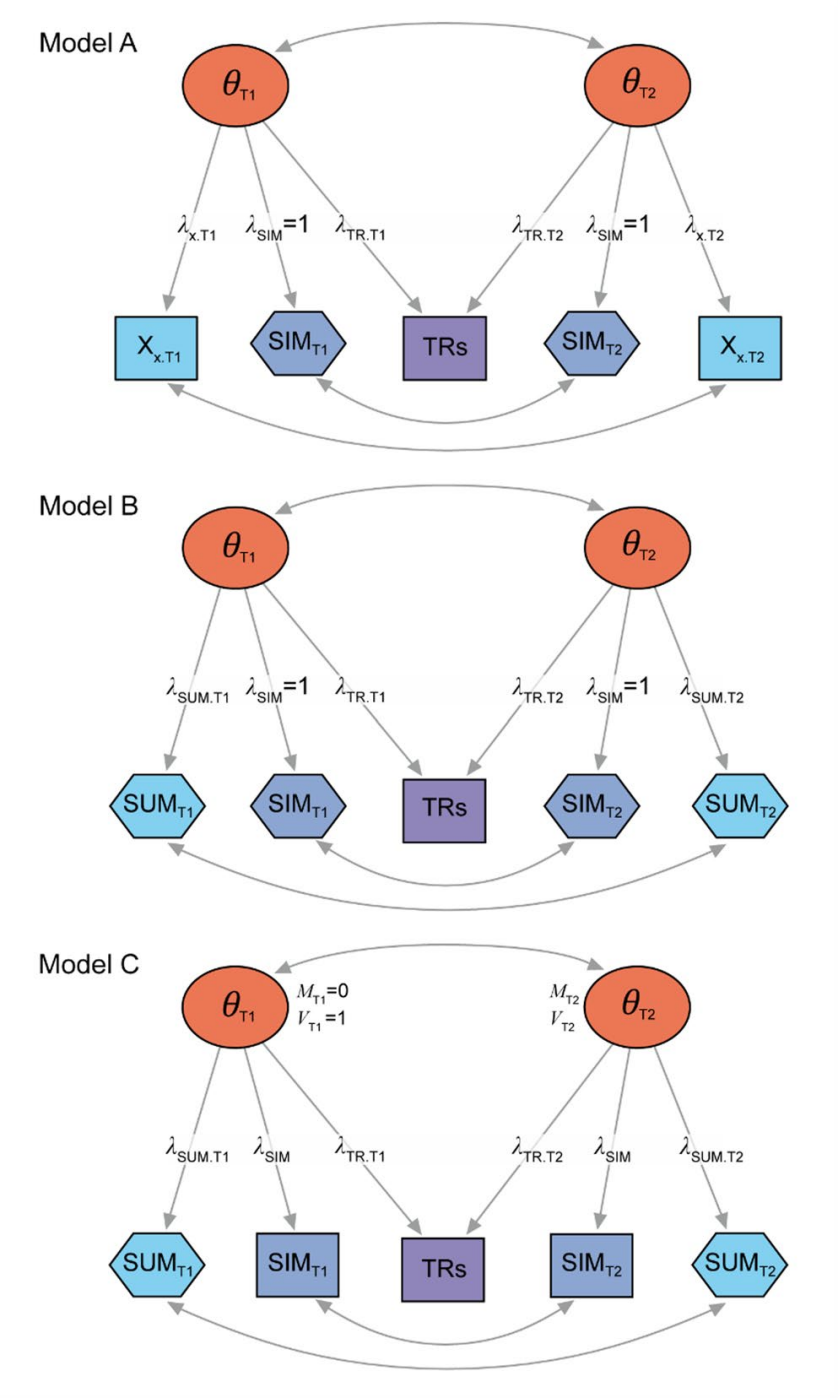
Three longitudinal confirmatory factor analysis (LCFA) models with auxiliary variables to estimate the MIC of a single item measure (SIM). Ovals represent latent constructs (factors, $\:\theta\:$) at baseline (T1) and follow-up (T2). Rectangles represent manifest variables treated as ordered indicators, and hexagons represent manifest variables treated as continuous indicators. The SIMs are indexed by time (T1 and T2). X indicates AUX items (indexed by the number of the item and the time). SUM indicates the AUX sum score (indexed by the time). TRs indicates the transition ratings item. *λ* represents factor loadings (indexed by the indicator, and time). *M* and *V* represent the mean and variance of $\:\theta\:$ at T1 and T2. Models A and B are identified by fixing the factor loadings of the SIMs. Model C is identified by standardizing $\:{\theta\:}_{\mathrm{T}1}$ and by constraining the factor loadings and thresholds of the ordinal SIMs to be the same over time. The factor loadings, intercepts/means and thresholds of the other indicators are freely estimated

We developed three LCFA-models of increasing complexity (Fig. 2). All models (A–C) were intended to recover the TRs reliability, necessary to calculate the APM-based MIC (MIC_APM_). Model C was specifically intended to recover the latent MIC, necessary to calculate the LCFA-based MIC (MIC_LCFA_). We used the R-package “lavaan”, version 0.6-17 [14] to perform the analyses.

- Model A is a correlated two-factor model, with the factors representing the latent construct of interest ($\:\theta\:$) at baseline ($\:{\theta\:}_{\mathrm{T}1}$) and follow-up ($\:{\theta\:}_{\mathrm{T}2}$) (Fig. 2). Indicators of $\:{\theta\:}_{\mathrm{T}1}$ are the SIM at baseline (SIM_T1_) and a randomly chosen AUX item at T1, indicators of $\:{\theta\:}_{\mathrm{T}2}$ are the SIM at follow-up (SIM_T2_) and the same AUX item at T2. The TRs item is included as an indicator of both time factors. Same indicators are allowed to correlate over time. The model is identified by fixing the factor loadings of the SIM variables ($\:{\lambda\:}_{\mathrm{S}\mathrm{I}\mathrm{M}}$) to 1. The SIM variables are treated as continuous indicators whereas the AUX item and the dichotomous TRs item are treated as ordered indicators. Diagonally weighted least squares (DWLS) was used to estimate the model, and scaled fit indices were used to evaluate model fit according to the Hu & Bentler criteria [15]. The reliability of the TRs is given by the model R^2^ value of the TRs item [10].
- Model B resembles model A, with the only difference being that the AUX sum score is used, instead of a single AUX item. The AUX sum score is treated as a continuous indicator.
- Model C resembles model B, with the difference being that the model is specified as a measurement invariance model for the SIM. Notably, the SIM variables are treated as ordered indicators, and the SIM factor loadings ($\:{\lambda\:}_{\mathrm{S}\mathrm{I}\mathrm{M}}$ in Fig. 2) and thresholds (not shown in Fig. 2) are constrained to be the same over time. The model is identified by standardizing $\:{\theta\:}_{\mathrm{T}1}$. The residual means and variances of the ordered indicators SIM_T1_ and TRs are set to 0 and 1 (theta parameterization), whereas the residual mean and variance of SIM_T2_ are freely estimated. The mean and variance of $\:{\theta\:}_{\mathrm{T}2}$ are also freely estimated. This specification allows for an accurate estimation of the latent change ($\:\varDelta\:\theta\:$) and trait at T2 ($\:{\theta\:}_{\mathrm{T}2}$). The latent MIC ($\:{\mathrm{M}\mathrm{I}\mathrm{C}}_{\theta\:}$) was calculated as $\:{\tau\:}_{\mathrm{T}\mathrm{R}\mathrm{s}}/{\lambda\:}_{\mathrm{T}\mathrm{R}\mathrm{s}.\mathrm{T}2}$, where $\:{\tau\:}_{\mathrm{T}\mathrm{R}\mathrm{s}}$ represents the threshold of the TRs item, and $\:{\lambda\:}_{\mathrm{T}\mathrm{R}\mathrm{s}.\mathrm{T}2}$ represents the factor loading of the TRs item on $\:{\theta\:}_{\mathrm{T}2}$ [8].

#### APM-based MIC estimation

The APM-based MIC was calculated using the following formula [6]:

$$\:{\mathrm{M}\mathrm{I}\mathrm{C}}_{\mathrm{A}\mathrm{P}\mathrm{M}}={\mathrm{M}\mathrm{I}\mathrm{C}}_{\mathrm{P}\mathrm{M}}-\left(0.8/{\mathrm{R}\mathrm{e}\mathrm{l}}_{\mathrm{T}\mathrm{R}\mathrm{s}}-0.5\right)\mathrm{*}\mathrm{S}\mathrm{D}\left(\varDelta\:\mathrm{S}\mathrm{I}\mathrm{M}\right)\mathrm{*}Cor\mathrm{*}\mathrm{log}\mathrm{o}\mathrm{d}\mathrm{d}\mathrm{s}\left(\mathrm{i}\mathrm{m}\mathrm{p}\right)$$

where $\:{\mathrm{R}\mathrm{e}\mathrm{l}}_{\mathrm{T}\mathrm{R}\mathrm{s}}$ represents the reliability of the TRs, $\:\mathrm{S}\mathrm{D}\left(\varDelta\:\mathrm{S}\mathrm{I}\mathrm{M}\right)$ represents the standard deviation of the SIM change score, $\:Cor$ represents the point biserial correlation between $\:\varDelta\:\mathrm{S}\mathrm{I}\mathrm{M}$ and the dichotomous TRs, $\:\mathrm{log}\mathrm{o}\mathrm{d}\mathrm{d}\mathrm{s}\left(\mathrm{i}\mathrm{m}\mathrm{p}\right)$ represents the log odds of the proportion improved, and $\:{\mathrm{M}\mathrm{I}\mathrm{C}}_{\mathrm{P}\mathrm{M}}$ represents the predictive modeling MIC [9]. The coefficients 0.8 and 0.5 have been derived through simulation studies [6].

#### LCFA-based MIC estimation

The LCFA-based MIC was calculated using the following formula:

$$\:{\mathrm{M}\mathrm{I}\mathrm{C}}_{\mathrm{L}\mathrm{C}\mathrm{F}\mathrm{A}}=\sqrt{\mathrm{v}\mathrm{a}\mathrm{r}\left({\mathrm{S}\mathrm{I}\mathrm{M}}_{\mathrm{T}1}\right)\mathrm{*}{\mathrm{R}\mathrm{e}\mathrm{l}}_{\mathrm{S}\mathrm{I}\mathrm{M}.\mathrm{T}1}}*{\mathrm{M}\mathrm{I}\mathrm{C}}_{\theta\:}$$

where $\:\mathrm{v}\mathrm{a}\mathrm{r}\left({\mathrm{S}\mathrm{I}\mathrm{M}}_{\mathrm{T}1}\right)$ represents the variance of $\:{\mathrm{S}\mathrm{I}\mathrm{M}}_{\mathrm{T}1}$, $\:{\mathrm{R}\mathrm{e}\mathrm{l}}_{\mathrm{S}\mathrm{I}\mathrm{M}.\mathrm{T}1}$ represents the reliability of $\:{\mathrm{S}\mathrm{I}\mathrm{M}}_{\mathrm{T}1}$, and $\:{\mathrm{M}\mathrm{I}\mathrm{C}}_{\theta\:}$ represents the latent MIC. $\:{\mathrm{R}\mathrm{e}\mathrm{l}}_{\mathrm{S}\mathrm{I}\mathrm{M}.\mathrm{T}1}$ was derived from the LCFA-model (model C) as the R^2^ statistic of $\:{\mathrm{S}\mathrm{I}\mathrm{M}}_{\mathrm{T}1}$. Note that [$\:\mathrm{v}\mathrm{a}\mathrm{r}\left({\mathrm{S}\mathrm{I}\mathrm{M}}_{\mathrm{T}1}\right)\mathrm{*}{\mathrm{R}\mathrm{e}\mathrm{l}}_{\mathrm{S}\mathrm{I}\mathrm{M}.\mathrm{T}1}$] represents the variance of the true score of $\:{\mathrm{S}\mathrm{I}\mathrm{M}}_{\mathrm{T}1}$ (i.e., the variance of $\:{\theta\:}_{\mathrm{T}1}$ in the metric of the SIM). $\:{\mathrm{M}\mathrm{I}\mathrm{C}}_{\theta\:}$ was calculated from the LCFA-model (model C, as described above).

#### Performance

The true MIC was simulated to be 0.8 (in the metric of the SIM) across all datasets (see Online Resource, Sect. 1). In each dataset, we tried to recover MIC_APM_ using LCFA-models A-C, and MIC_LCFA_ using LCFA-model C. The performance of the methods and models with respect to recovering the true MIC, were evaluated through calculating the bias (i.e., the difference between the mean estimates and the true value) and the root mean squared error (RMSE, the square root of the mean of the squared differences between the estimates and the true value), using the R-package “rsimsum”, version 0.11.3 [18]. Additionally, we explored any impact of the simulation parameters on the bias in the estimates through multivariable linear regression, regressing bias (i.e., estimate – true value) on the parameters and their two- and three-way interactions. Backward elimination was used to remove little contributing parameters and interactions (explaining < 2% of the bias variance; 2% was—somewhat arbitrarily—chosen to obtain well interpretable final regression models with a limited number of “predictors”).

### Real data

#### Patients and measurements

To illustrate the proposed approaches to the MIC of SIMs, we analyzed PROM data from a cohort of patients (*n* = 411) undergoing knee replacement surgery in the Jeroen Bosch Hospital, ‘s-Hertogenbosch, the Netherlands, between February 1st 2021 and August 1st 2023. Before surgery and six months later, patients completed an NRS for pain during activities (the SIM of interest) and another NRS for pain at rest. The NRS for pain during activities read “How much knee pain (affected side) have you experienced during activities during the past week?”. Patients could respond by ticking an 11-point response scale from 0 to 100 in steps of 10. Furthermore, they completed the Oxford Knee Score (OKS), a 12-item questionnaire measuring knee pain and related functional problems during the past four weeks [16]. The items have 5 response options. The OKS consists of two subscales, pain and function [17]. We used the pain subscale. At 6 months, patients also completed a TRs question (“How has your pain changed since the surgery? Very much deteriorated, Much deteriorated, A little deteriorated, Not changed, A little improved, Much improved, Very much improved”). If necessary, scores were reverse-coded so that all scores were in the same direction: higher is better.

#### Analyses

The SIM of interest was linearly transformed by dividing the scores by 10, obtaining an integer NRS scale ranging from 0 to 10. After the estimation of MIC on the transformed SIM scale, the MIC was back-transformed to the original SIM scale by multiplying the value by 10. The AUXs were not transformed. We dichotomized the TRs into “improved” (Much improved and Very much improved) and “not-improved” (Much deteriorated, A little deteriorated, Not changed, and A little improved). We chose the threshold between “A little improved” and “Much improved” on the anchor because almost all patients indicated to have experienced at least a “little” improvement. As such, the threshold may be of a larger magnitude than “minimal”, but nevertheless it illustrates the ability of the method to estimate a threshold of meaningful improvement. We used the OKS pain subscale score as AUX in a model C type LCFA-model to estimate the TRs reliability (for calculating MIC_APM_) and the latent MIC (for calculating MIC_LCFA_). Non-parametric bootstrapping (2000 replications) was used to estimate 95% confidence intervals (95% CI). To assess the robustness of the results, we repeated the analysis using the NRS pain at rest as AUX.

## Results

### Simulations

#### Sample characteristics and model convergence

Table 2 provides an overview of the sample characteristics. The simulated datasets varied widely. In some datasets the AUX sum scores, and to a lesser extent the SIM scores, were skewed, especially at T2. The correlation between the SIM scores and the AUX sum scores varied between 0.28 and 0.89. The biserial correlation between the SIM change score and the TRs varied between 0.11 and 0.77.

**Table 2:** Sample characteristics across 15,552 simulated datasets

Characteristic	Mean (range)
Mean SIM T1 score	5.50 (4.44, 6.58)
SD SIM T1 score	1.30 (1.11, 1.51)
Skewness SIM T1 score	0.00 (− 0.22, 0.20)
Kurtosis SIM T1 score	− 0.16 (− 0.62, 0.37)
Floor effects^a^ SIM T1 score	0.01 (0.00, 0.04)
Ceiling effects^b^ SIM T1 score	0.01 (0.00, 0.03)
Mean SIM T2 score	6.29 (4.27, 8.30)
SD SIM T2 score	1.45 (1.04, 1.89)
Skewness SIM T2 score	− 0.08 (− 0.77, 0.24)
Kurtosis SIM T2 score	− 0.16 (− 0.62, 0.37)
Floor effects SIM T2 score	0.01 (0.00, 0.07)
Ceiling effects SIM T2 score	0.04 (0.00, 0.27)
Mean SIM change score	0.79 (− 0.27, 1.86)
SD SIM change score	1.38 (1.01, 1.80)
Skewness SIM change score	− 0.02 (− 0.33, 0.20)
Kurtosis SIM change score	− 0.03 (− 0.39, 0.43)
Floor effects SIM change score	0.00 (0.00, 0.03)
Ceiling effects SIM change score	0.00 (0.00, 0.03)
Reliability* AUX T1 score	0.84 (0.76, 0.90)
Mean AUX T1 score	15.0 (8.4, 21.5)
SD AUX T1 score	6.6 (5.5, 8.0)
Skewness AUX T1 score	0.01 (− 0.85, 0.85)
Kurtosis AUX T1 score	− 0.49 (− 1.05, 0.20)
Floor effects AUX T1 score	0.01 (0.00, 0.09)
Ceiling effects AUX T1 score	0.01 (0.00, 0.10)
Mean AUX T2 score	19.5 (7.5, 28.2)
SD AUX T2 score	6.5 (2.8, 9.8)
Skewness AUX T2 score	− 0.61 (− 2.80, 0.96)
Kurtosis AUX T2 score	0.32 (− 1.33, 10.90)
Floor effects AUX T2 score	0.01 (0.00, 0.19)
Ceiling effects AUX T2 score	0.10 (0.00, 0.50)
Mean AUX change score	4.5 (− 2.2, 12.2)
SD AUX change score	6.5 (4.6, 9.1)
Skewness AUX change score	0.02 (− 0.44, 0.74)
Kurtosis AUX change score	0.01 (− 0.68, 1.23)
Floor effects AUX change score	0.00 (0.00, 0.00)
Ceiling effects AUX change score	0.00 (0.00, 0.00)
Correlation** SIM_T1_ – AUX_T1_	0.59 (0.35, 0.82)
Correlation** SIM_T2_ – AUX_T2_	0.61 (0.28, 0.89)
Correlation*** ΔSIM - TRs	0.46 (0.11, 0.77)

SIM, single item measure; T1, baseline; T2, follow-up; AUX, auxiliary patient-reported outcome measure (sum score); SD, Standard deviation; ΔSIM, SIM change score; TRs, transition ratings; ^a^Floor effect, proportion of simulees in the lowest score category of the scale; ^b^Ceiling effect, proportion of simulees in the highest score category of the scale; *Cronbach’s alpha, **Pearson correlation, ***Biserial correlation

In each of the 15,552 datasets, three different LCFA-models (Fig. 2) were fitted and the MIC_APM_ was calculated three times (using the TRs reliabilities from models A–C) whereas MIC_LCFA_ was calculated once (using the latent MIC from model C). Eight hundred and two LCFA-models (1.7% of the total number of 46,656 models estimated) did not converge. Model A failed to converge in 2.7% of the datasets, model B failed in 0.9%, and model C failed in 1.5%. The comparative fit index (CFI) indicated poor fit in none of the converged models (poor fit defined as CFI < 0.95; smallest CFI: 0.988), the Tucker-Lewis index (TLI) indicated poor fit in 0.26% of the models (poor fit defined as TLI < 0.95; smallest TLI: 0.875), the root mean square error of approximation (RMSEA) indicated poor fit in 0.33% of the models (poor fit defined as RMSEA > 0.06; highest RMSEA: 0.139), and the standardized root mean squared residual (SRMR) indicated poor fit in none of the models (poor fit defined as SRMR > 0.08; highest SRMR: 0.033).

#### MIC estimates

**Fig. 3:**
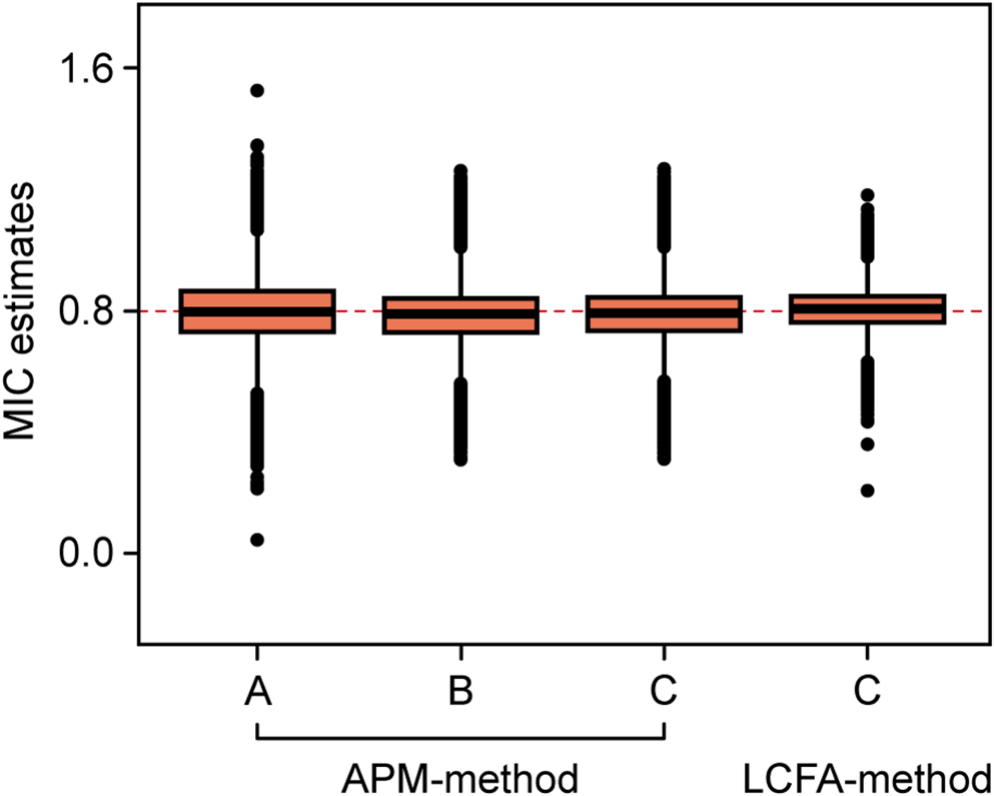
Distribution of MIC estimates by MIC-estimation method (APM and LCFA) and LCFA-model (A–C) used. The true simulated MIC was 0.8 (dashed line). APM, adjusted predictive modeling; LCFA, longitudinal confirmatory factor analysis

The bias and variability of the MIC estimates, based on the APM- and LCFA-methods (and the various LCFA-models), is shown in Table 3. In all cases the bias was negligible. Figure 3 shows that the APM-method recovered the true MIC well using LCFA-models B and C (and to a slightly lesser extent, model A), and that the LCFA-method correctly recovered the true MIC using model C. Table 3 also shows that the LCFA-method slightly outperformed the APM-method with respect to the precision (RMSE) of the MIC estimates. Regression analysis of MIC_APM_ on the simulation parameters revealed that the combination of high PSB and high or low proportion improved was largely responsible for this difference in precision between the methods (see Online Resource, Sect. 3.1 through 3.3). We checked the ability of the LCFA-models to recover the TRs reliability (see Online Resource, Sect. 4). In addition, model C proved to accurately recover the latent MIC (data not shown).

**Table 3:** Performance of the MIC Estimation methods (APM and LCFA) by the LCFA-model (A–C) used

Performance measure	APM-method			LCFA-method
	Model A	Model B	Model C	Model C
N	15,131	15,411	15,312	15,312
Mean estimate	0.796	0.785	0.789	0.803
Bias	− 0.004 (0.001)	− 0.016 (0.001)	− 0.011 (0.001)	0.003 (0.001)
RMSE	0.168 (0.019)	0.152 (0.018)	0.154 (0.018)	0.072 (0.009)

N, number of samples/converged models; RMSE, root mean squared error

APM, adjusted predictive modeling; LCFA, longitudinal confirmatory factor analysis

The true MIC was 0.8. Values between brackets are Monte Carlo standard errors

### Real data

There were 240 woman and 171 men, their mean age was 71.5 (range 25–89). The mean SIM score improved from 24.3 at baseline to 71.2 six months later (Table 4). The proportion improved was 0.83, and the point-biserial correlation between the anchor and the SIM change score was 0.48.

**Table 4:** Real data sample statistics

Statistic		Estimate
Gender F/M		240/171
Age, mean (SD)		71.5 (9.0)
SIM_T1_ score, mean (SD)*		24.3 (18.5)
SIM_T2_ score, mean (SD)*		71.2 (25.1)
Proportion improved		0.83
TRs-SIM change correlation**		0.48
TRs-response, numbers (%)		
Very much deteriorated		5 (1.2%)
Much deteriorated		9 (2.2%)
A little deteriorated		3 (0.7%)
Not changed		17 (4.1%)
A little improved		36 (8.7%)
Much improved		180 (43.9%)
Very much improved		161 (39.2%)

SIM, single item measure; T1, baseline; T2, follow-up; TRs, transition ratings

*Reverse coded (higher = better)

**Point-biserial correlation

Table 5 shows the results of the analyses. Considerable numbers of LCFA-models in the bootstrapped datasets did not converge, 35.7% of the models failed to converge if the OKS pain subscale was used as AUX, and even 59.1% of the models failed if the NRS pain at rest was used. The analyses using different AUXs produced fairly comparable results. MIC_APM_ was estimated to be 21.0 and 20.9 for OKS pain subscale and NRS pain at rest as AUX respectively, whereas MIC_LCFA_ was estimated to be 17.0 and 16.9 (Table 5). Given the high proportion improved (83%) and the high degree of PSB, MIC_APM_ was probably an overestimation of the true MIC (see Online Resource, Sect. 3.3). Hence, MIC_LCFA_ should be considered as the most trustworthy estimate of the MIC (i.e., as the best estimate of the mean individual MIC). However, the difference between the MICs was relatively small (4 points) given the 100-point scale of the SIM, and also given the 95% CIs of the MIC estimates.

**Table 5:** Results of the real data

Statistic	Auxiliary variable	
	OKS pain subscale	NRS pain at rest
Number of converged bootstrap samples	1287	818
SIM-AUX correlation T1	0.58 (0.47, 0.65)	0.62 (0.53, 0.68)
SIM-AUX correlation T2	0.78 (0.70, 0.82)	0.78 (0.71, 0.83)
CFI	0.970 (0.923, 1)	0.968 (0.906, 1)
TLI	0.963 (0.904, 1)	0.960 (0.887, 1)
RMSEA	0.096 (0, 0.141)	0.095 (0, 0.144)
SRMR	0.009 (0.001, 0.033)	0.007 (0, 0.028)
Mean latent change	2.25 (1.98, 3.35)	2.78 (1.90, 3.65)
SD latent change	1.29 (1.09, 1.69)	1.49 (1.08, 1.97)
$\:{\mathrm{R}\mathrm{e}\mathrm{l}}_{\mathrm{T}\mathrm{R}\mathrm{s}}$	0.678 (0.641, 0.927)	0.676 (0.478, 0.856)
PSB	0.871 (0.594, 0.990)	0.812 (0.534, 1.036)
$\:{\mathrm{M}\mathrm{I}\mathrm{C}}_{\theta\:}$	0.98 (0.88, 1.73)	1.20 (0.58, 1.69)
$\:{\mathrm{R}\mathrm{e}\mathrm{l}}_{\mathrm{S}\mathrm{I}\mathrm{M}.\mathrm{T}1}$	0.899 (0.461, 1)	0.589 (0.419, 1)
MIC_APM_	21.0 (19.5, 28.6)	20.9 (12.6, 27.2)
MIC_LCFA_	17.0 (14.8, 22.1)	16.9 (9.3, 20.5)

OKS, Oxford Knee Score, NRS, numeric rating scale; SIM, single item measure; AUX, auxiliary variable; T1, baseline; T2, follow-up; CFI, comparative fit index; TLI, Tucker-Lewis index; RMSEA, root mean square error of approximation; SRMR, standardized root mean squared residual; Rel, reliability; TRs, transition ratings; PSB, present state bias; MIC, minimal important change; $\:{\mathrm{M}\mathrm{I}\mathrm{C}}_{\theta\:}$, latent MIC; APM, adjusted predictive modeling; LCFA, longitudinal confirmatory factor analysis

## Discussion

### Summary and interpretation of findings

In simulation studies, both the APM-method (using any of the LCFA-models A-C) and the LCFA-method (using model C) proved to recover the true MIC. Nevertheless, there were differences in precision. The LCFA-method appeared to be slightly more precise than the APM-method. Indeed, in the real data we observed a somewhat higher precision of the LCFA-method (a smaller width of the 95% CI). Moreover, the APM-method using model B or C was slightly more precise than the same method using model A. We assume that this was due to the sum score of several items providing more “information” than a single item with a limited number of response options. This difference in “information” may also explain the difference in precision between the real data MIC estimates when the OKS pain subscale score was used as AUX, compared to when the NRS pain in rest was used.

### How to deal with continuous sims

The use of LCFA-model C (to recover either the TRs reliability or the latent MIC) requires that the SIM is a categorical variable with a limited number of ordered response categories.^3^ However, some SIMs, such as a VAS or a digital health measure, are continuous in nature. Continuous variables can be dealt with through linear transformation and discretization. The first step is to multiply the variable by a factor T = 11.98/(range of the scale). The second step is to add a factor *R* = 0.51 – (minimum value of the transformed scale). These steps transform the variable into one ranging from 0.51 to 12.49. The third step is to round the values to integers, which results in a 12-point ordinal scale that can be used in LCFA-model C. After the calculation of the MIC on the transformed scale, the MIC needs to be multiplied by 1/T to obtain the MIC on the original scale.

### Limitation

Across the simulations, we let the SIM and the TRs target the same construct ($\:\theta\:$). This assumption is realistic because, in general, we are interested in estimating the MIC of a construct that an SIM is measuring, and the patients’ evaluation of change should concern that same construct. If the TRs target a “broader” or “narrower” construct than that of the SIM, the MIC estimate may be biased. More work needs to be undertaken to understand the limit of the construct agreement between the TRs and the SIM and to characterize the bias when the two do not align.

LCFA-models with no more than the minimally required three indicators per factor (where one indicator also serves as an indicator of two factors), contain relatively little “information” for the statistical procedure to recover the latent construct, rendering these models vulnerable for non-convergence. This was illustrated by the substantial prevalence of non-convergence in the bootstrap replications of the real data. In the simulations this was a much smaller problem due to the large sample size of the simulated datasets. The addition of more AUX variables to the LCFA-model would certainly be beneficial for model convergence. However, when the SIM and TRs variables (that target the construct of interest) become numerically dominated by the AUX variables (that target only part of that construct), the modeled latent construct will meaningfully differ from the latent construct of interest, resulting in biased estimates of the TRs reliability and the latent MIC. Our simulations have shown that an LCFA-model with only one AUX variable that does not quite target the construct of interest, next to two variables that do target that construct, is able to recover the latent construct of interest.

### Conclusions and recommendations

Estimating an MIC of an SIM can be done with the APM-method [6] or the LCFA-method [8] when an AUX is available, that correlates with the SIM. Both methods require the estimation of an LCFA-model to obtain either the TRs reliability (to calculate MIC_APM_), or the latent MIC (to calculate MIC_LCFA_). Greater degrees of PSB (i.e., > 0.4) in combination with relatively high (i.e., > 0.6) or low (i.e., < 0.4) proportions improved tend to bias MIC_APM_ but not MIC_LCFA_. This makes the LCFA-method the preferred method. The APM-method is a feasible alternative, when the proportion improved is not too high or too low, or when the PSB is limited. Only model C allows for the correct estimation of PSB. Model C is characterized by a single AUX indicator *and* measurement invariance constraints on the SIMs. The AUX indicator can be the sum score of any number of AUX items.

We recommend to always estimate the precision of an MIC estimate. Nonparametric bootstrapping is a feasible method (the R-code used for the real dataset is given in Online Resource, Sect. 8). The customary reporting of 95% CIs will hopefully prevent the publication of grossly imprecise MIC estimates based on too small sample sizes.

This work has focused on two relatively new MIC estimation methods that both require the application of advanced statistical techniques (i.e., LCFA). Is there still room for “older” MIC estimation methods that do not require LCFA, such as mean change [18], ROC-analysis [19], predictive modeling [9], or the “old” APM-method [5]? We don’t think there is. If the goal of MIC estimation is to recover the mean of individual MICs, only the newer methods suffice. Just to illustrate our position, we have used the “best” of the older methods, the “old” APM-method [5], to estimate an MIC in our simulated datasets (see Online Resource, Sect. 5). The “old” MIC_APM_ was significantly less precise than the newer methods, being biased by the proportion improved and other sample characteristics. Although the application of LCFA represents a high point of entry for using the newer methods, the benefit is that estimates are less biased and less variable compared to the older methods. Therefore, researchers should prioritize the use of these methods in their MIC studies. To support research teams in this, the R-code provided in the Online Resource can be adapted for use.

## Supplementary Information

Below is the link to the electronic supplementary material.

Supplementary Material 1

## Data Availability

The R-code used to simulate and analyze samples is provided in the Supplementary material. The real data is available on reasonable request from Babette van der Zwaard (bvanderzwaard@yahoo.com).

## Footnotes

1 The change can occur in two directions, improvement and deterioration. For simplicity, we will focus only on improvement.

2 One of the reviewers suggested the analysis of more than one value for the true MIC. We repeated a limited analysis of 1296 datasets with true MIC values of 0.5 and 1.0. The results, shown in Online Resource, Sect. 6, echoed those for a true MIC of 0.8.

3 The statistical program lavaan accepts items with up to 12 ordered response categories.

## Code availability

The R-code is provided in the Supplementary material.
