# Supplementary material for: Estimating the minimal important change of single-item measures using the adjusted predictive modeling method or the longitudinal confirmatory factor analysis method
Source: Qual Life Res. 2026 Jan 9;35(2):39. doi: 10.1007/s11136-025-04134-3 (PMC12789162; doi:10.1007/s11136-025-04134-3)
Supplement: Supplementary file 1 — Supplementary Material 1 [file 11136_2025_4134_MOESM1_ESM.docx]

Online Supplement

**Estimating the minimal important change of single-item measures using the adjusted predictive modeling method or the longitudinal confirmatory factor analysis method**

Berend Terluin, Yong Hao Pua, Piper Fromy, Andrew Trigg, Babette van der Zwaard, Jakob B Bjorner

Quality of Life Research

**Content**

[1. Data simulation 2](#_Toc196384155)

[2. Item parameters for simulation 5](#_Toc196384156)

[3. What determines the variability of the MIC estimates? 6](#_Toc196384157)

[3.1. Adjusted predictive modeling MIC, model A 6](#_Toc196384158)

[3.2. Adjusted predictive modeling MIC, model B 7](#_Toc196384159)

[3.3. Adjusted predictive modeling MIC, model C 8](#_Toc196384160)

[3.4. LCFA-based MIC, model C 9](#_Toc196384161)

[4. Recovering the transition ratings reliability 10](#_Toc196384162)

[5. Old adjusted predictive modeling MIC 11](#_Toc196384163)

[6. Alternative true MIC values 13](#_Toc196384164)

[7. R-code for simulation and estimation 15](#_Toc196384165)

[8. R-code for the MIC of single-item measures 33](#_Toc196384166)

# 1. Data simulation

*SIM response data*

Fig. S1.1 presents a graphical outline of the data simulation process. As an example of an SIM, we simulated responses to a numeric rating scale (NRS) with a score range from 1 to 10, administered at baseline (T1) and follow-up (T2), for samples of 2000 simulated patients (“simulees”). We started to simulate two uncorrelated random variables to represent two components of the baseline (T1) latent trait of the construct of interest ($\theta_{T1}$ in Fig. S1.1), components A and B ($\theta_{T1}^{A}$ and $\theta_{T1}^{B}$ in Fig. S1.1). $\theta_{T1}^{A}$ is the component underlying both the SIM and the AUX scores, and is the component that is responsible for the correlation between the SIM and the AUX. $\theta_{T1}^{B}$ is a component that is unique for the SIM. We varied the variance of $\theta_{T1}^{B}$ between nil and equal to $\theta_{T1}^{A}$. The mean and variance of $\theta_{T1}^{A}$ and $\theta_{T1}^{B}$ were simulated in such a way that the construct of interest ($\theta_{T1}$, i.e., the sum of $\theta_{T1}^{A}$ and $\theta_{T1}^{B}$) had a standard deviation (SD) of 1 and a mean of -1, 0 or 1 (see Table 1 for an overview of the simulation parameters that were varied across the simulations). Furthermore, we created two variables representing the change in the latent components between baseline and follow-up (${\Delta\theta}^{A}$ and ${\Delta\theta}^{B}$ in Fig. S1.1). The means and variances of the change components were simulated in such a way that the latent change in the construct of interest ($\Delta\theta$ [not shown in Fig. S1.1], i.e., the sum of ${\Delta\theta}^{A}$ and ${\Delta\theta}^{B}$) had a varying mean (to create different proportions improved) and a varying SD (to create more or less heterogeneity in the latent change). The latent change (components) could be correlated with the baseline trait (components) or not (*R*1 in Fig. S1.1). The latent trait (components) of the construct of interest at follow-up ($\theta_{T2}$ in Fig. S1.1) was calculated by summing the latent baseline trait components and the corresponding latent change components (i.e., $\theta_{T2}^{A}=\theta_{T1}^{A}+{\Delta\theta}^{A}$, $\theta_{T2}^{B}=\theta_{T1}^{B}+{\Delta\theta}^{B}$, and $\theta_{T2}=\theta_{T2}^{A}+\theta_{T2}^{B}$).

Next, the latent traits underlying the SIM scores ($\mathrm{SIM}_{T1}^{*}$ and $\mathrm{SIM}_{T2}^{*}$ in Fig. S1.1) were created by adding to $\theta_{T1}$ and $\theta_{T2}$ two variables ($\eta_{T1}$ and $\eta_{T2}$ in Fig. S1.1), representing a unique construct underlying the SIM scores, with a correlation of 0.5 or 0.9 (*R*2 in Fig. S1.1). In addition, two random variables representing measurement error were added (*E*1 and *E*2 in Fig. S1.1). $\eta$ accounts for the SIM possibly measuring, apart from the construct of interest, something different, for instance the tendency to choose relatively high of low scores on the SIM scale. $\eta$ and the error variables were given means of zero and SDs to create a reliability of the SIM score of 0.5 or 0.8. Note that, with respect to the reliability of the SIM as an instrument to measure $\theta$, $\eta$ counts as error. For simplicity, we divided the variance of the total error per SIM equally across *E*1 and $\eta_{T1}$ (and across *E*2 and $\eta_{T2}$ respectively). So, the latent traits underlying the manifest SIM scores ($\mathrm{SIM}_{T1}^{*}$ and $\mathrm{SIM}_{T2}^{*}$) consisted of $\theta_{T1}$, $\eta_{T1}$ and *E*1, and $\theta_{T2}$, $\eta_{T2}$ and *E*2 respectively. The final step was to apply a set of 9 thresholds (from -4 to +4 in steps of 1; *Thr*1 in Fig. S1.1) to arrive at the manifest SIM scores ($\mathrm{SIM}_{T1}$ and $\mathrm{SIM}_{T2}$ in Fig. S1.1), i.e., the NRS scores in the range of 1 to 10. Simulees with a latent $\mathrm{SIM}^{*}$ score ≤ -4 were given a manifest SIM score of “1”, simulees with a latent $\mathrm{SIM}^{*}$ score between -4 and -3 were given a manifest SIM score of “2”, …, simulees with a latent $\mathrm{SIM}^{*}$ score > +4 were given a manifest SIM score of “10”. It should be noted that this way of simulating manifest SIM scores provides a direct link between the metric of the latent trait ($\theta$) of interest and the metric of the SIM. Merely for reasons of simplicity, we chose to let one unit of the $\theta$ scale (which equals the SD of $\theta_{T1}$) to correspond to one SIM point. Therefore, whereas the true MIC in terms of the $\theta$ metric was set to be 0.8 across the simulated datasets (see below), the true MIC in terms of the change in SIM score was also 0.8.


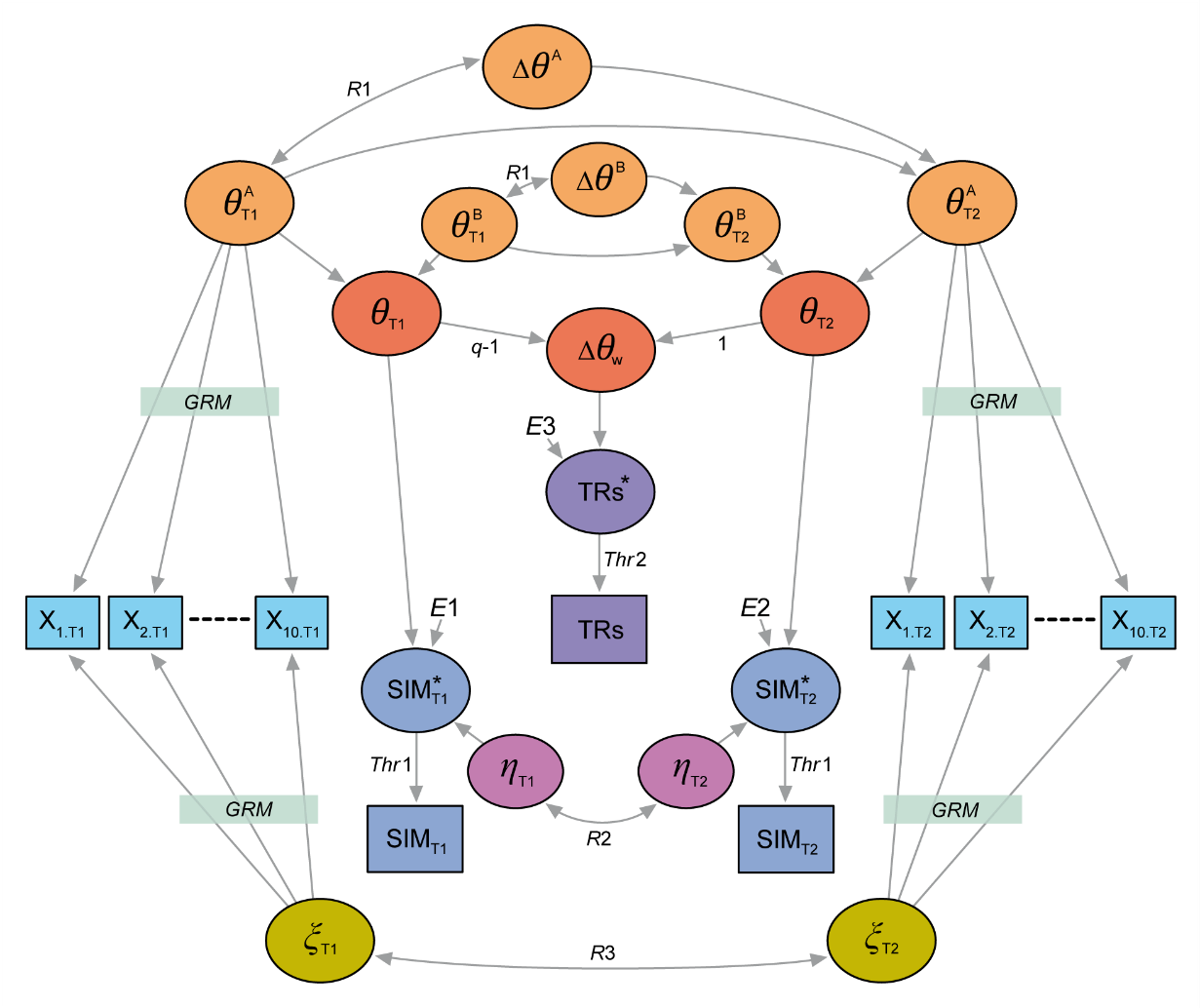


**Fig. S1.1** Graphical outline of the data simulation process. Ovals represent latent constructs at baseline (T1) and follow-up (T2), and their change ($\Delta$). Rectangles represent manifest variables: single-item measure (SIM) scores (at T1 and T2), multi-item AUX (X) scores (at T1 and T2) and transition ratings (TRs). $\theta$ represents the construct of interest underlying the SIM scores and the TRs, and partially underlying the X scores, with components $\theta^{A}$ and $\theta^{B}$. $\xi$ represents a second construct uniquely underlying the X scores. $\eta$ represents a second construct uniquely underlying the SIM scores. SIM* represents the latent trait underlying the manifest SIM scores (at T1 and T2). TRs* represents the “perceived change”, the latent trait underlying the transition ratings (TRs). $q$ (0 ≤ $q$ ≤ 1) represents the degree of present state bias. ${\Delta\theta}_{w}$ represents the weighted change, underlying the “perceived change” (TRs*). *E* represents (measurement) error. *Thr* represents a set of thresholds (*Thr*1) or a single threshold (*Thr*2). *R* represents correlations. *GRM* refers to the graded response model used to generate the AUX scores (X_1.T1_ … X_10.T2_) based on the underlying latent constructs ($\theta^{A}$ and $\xi$) and a set of item parameters (not shown).

*AUX item response data*

In order to simulate AUX item response data using item response theory (IRT), we created a set of item parameters (i.e., slope and location parameters) for a hypothetical PROM with 10 items with four response options (see the Online Resource, Sect. 2). Furthermore, we simulated two unique latent factors, at baseline and follow-up ($\xi_{T1}$ and $\xi_{T2}$ in Fig. S1.1) with a correlation of 0.5 or 0.9 (*R*3 in Fig. S1.1). The $\theta^{A}$ and $\xi$ factors were uncorrelated. We generated item response data based on the graded response model (*GRM* in Fig. S1.1). The AUX item responses (X_1.T1_ to X_10.T2_ in Fig. S1.1) were based on a two-factor model with factors $\theta^{A}$ and $\xi$ at T1 and T2, and the item parameters mentioned above. The slope parameters for $\xi$ were the same as for $\theta^{A}$, but randomly reordered over the items, and multiplied by a factor between 0 and 0.5 (see Table 1) in order to reduce the strength of $\xi$, relative to the strength of $\theta^{A}$.

*Transition ratings data*

TRs were based on the “perceived change” (TRs* in Fig. S1.1), whereas the perceived change consisted of the “weighted change” (${\Delta\theta}_{w}$ in Fig. S1.1) and measurement error (*E*3 in Fig. S1.1). The weighted change takes into account a variable degree of “present state bias” (PSB), the phenomenon that TRs often are more heavily influenced by the follow-up state ($\theta_{T2}$) than by the baseline state ($\theta_{T1}$).^[[1]](#footnote-1),^^[[2]](#footnote-2)^ The weighted change was calculated using the following formula:

${\Delta\theta}_{w}=q*(\theta_{T2}-\bar{\theta_{T1}})+\left( 1-q \right)*\Delta\theta$

where $q$ represents a variable of weights between 0 and 1, indicating the degree of PSB (0 = no PSB; 1 = complete PSB), and where $\bar{\theta_{T1}}$ is the mean of the baseline state ($\theta_{T1}$).^[[3]](#footnote-3)^ We varied $q$ between 0 and 0.8 (Table 1). Measurement error (*E*3 in Fig. S1.1) was added to the weighted change as a random variable to obtain the perceived change. The error variable had a mean of zero and an SD calculated to create a reliability of the TRs of 0.4 or 0.7 (Table 1). To obtain manifest TRs (TRs in Fig. S1.1), we created a variable of individual MICs with an (arbitrary) mean of 0.8 and an (arbitrary) SD of 0.075. Simulees whose perceived change (TRs*) exceeded their individual MIC were rated as “improved”, otherwise they were rated as “not-improved”.

# 2. Item parameters for simulation^[[4]](#footnote-4)^

Item response theory (IRT) provides a probabilistic model for understanding persons’ responses to the items of a PROM as an interaction between person characteristics and item characteristics. The person characteristic of interest is their standing on the construct the PROM is purported to measure (e.g., the person’s level of physical functioning). Relevant item characteristics are ‘slope’ and ‘location’. In the graded response model, the slope parameter (α) reflects the strength of an item as an indicator of the construct. The location parameter (β) is a characteristic of the item’s response options, and reflects the level of the construct at which the probability to endorse a particular response option (or a higher option) equals 50%. For items with 4 response options there are 3 location parameters (β1, β2, β3).

The item parameters were simulated as follows: The β2 parameters consisted of a series of numbers between -0.8 and +0.8 with intervals of 0.4. The β1 parameters were based on β2 minus 1 plus a random distribution of β2/4. The β3 parameters were based on β2 plus 1 plus a different random distribution of β2/4. The α parameters consisted of 1.7 + β2/2, randomly distributed. Table S2.1 shows the parameters.

**Table S2.1**. Item parameters of hypothetical questionnaire with 10 items, reliability ~0.86

| **Item** | **α** | **β1** | **β2** | **β3** |
| --- | --- | --- | --- | --- |
| 1 | 2.1 | -1.9 | -0.8 | 0.4 |
| 2 | 1.5 | -1.7 | -0.8 | 0.0 |
| 3 | 1.3 | -1.6 | -0.4 | 0.7 |
| 4 | 1.9 | -1.4 | -0.4 | 0.7 |
| 5 | 1.7 | -0.8 | 0.0 | 1.0 |
| 6 | 1.9 | -0.8 | 0.0 | 1.2 |
| 7 | 2.1 | -0.5 | 0.4 | 1.3 |
| 8 | 1.5 | -0.6 | 0.4 | 1.2 |
| 9 | 1.7 | -0.3 | 0.8 | 1.7 |
| 10 | 1.3 | -0.4 | 0.8 | 1.8 |
| **Mean** | **1.7** | **-1.0** | **0.0** | **1.0** |

# 3. What determines the variability of the MIC estimates?

## 3.1. Adjusted predictive modeling MIC, model A

All simulation parameters and their 2- and 3-way interactions explained 73.1% of the MIC variability. After backward elimination a final model remained with 4 predictors, the PSB, the SD of the latent change, the correlation between the latent baseline trait and the latent change, and the proportion improved, explaining 70.9% (adjusted R^2^) (Box S3.1).

**Box S3.1** Regression model for MIC_APM_, model A

Coefficients:

Estimate Std. Error t value Pr(>|t|)

(Intercept) 0.794702 0.001371 579.44 <2e-16 ***

psb.par -0.628888 0.004108 -153.10 <2e-16 ***

sd.tetchs.par:cor.t1.ch.par -0.246558 0.005777 -42.68 <2e-16 ***

psb.par:prop.imp.par 1.250651 0.006871 182.03 <2e-16 ***

sd.tetchs.par:cor.t1.ch.par:prop.imp.par 0.470768 0.010019 46.99 <2e-16 ***

---

Signif. codes: 0 ‘***’ 0.001 ‘**’ 0.01 ‘*’ 0.05 ‘.’ 0.1 ‘ ’ 1

Multiple R-squared: 0.7089, Adjusted R-squared: 0.7088

F-statistic: 9207 on 4 and 15126 DF, p-value: < 2.2e-16

The most important predictors were the PSB and the proportion improved, together explaining 67.7% of the variability in the MIC estimates. Fig. S3.1 shows how MIC_APM_ was biased by the PSB in combination with the proportion improved. We have seen this effect in a previous study.^[[5]](#footnote-5)^


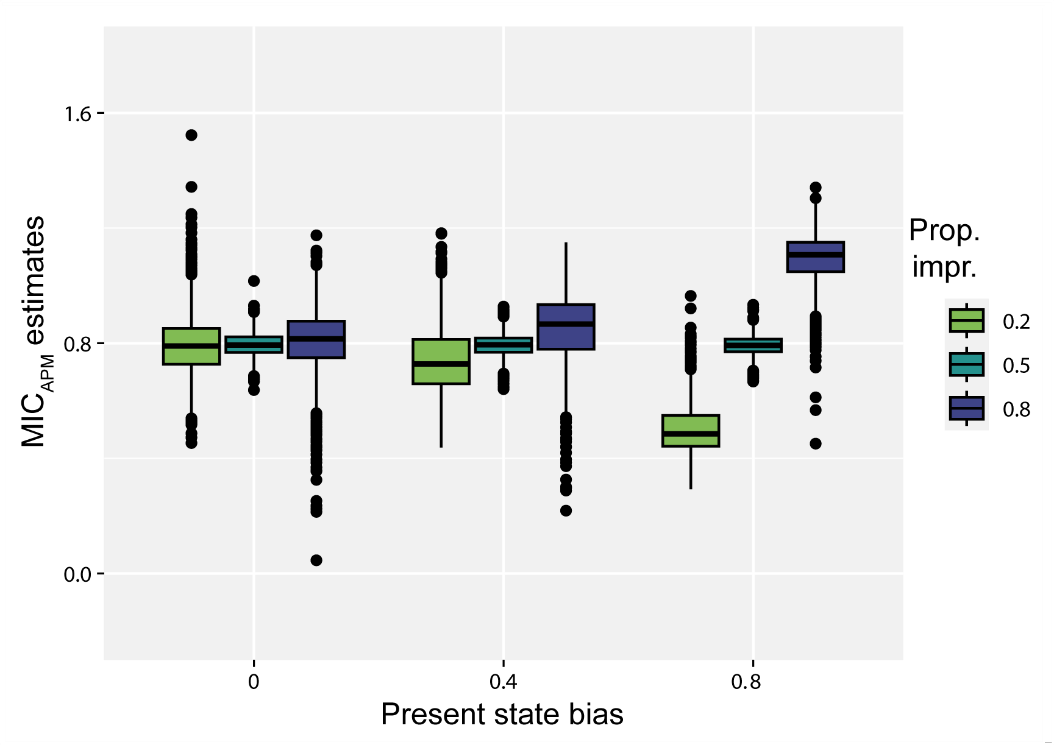


**Fig. S3.1** Distribution of MIC_APM_ (model A) estimates by the present state bias and the proportion improved. The true MIC was 0.8.

## 3.2. Adjusted predictive modeling MIC, model B

All simulation parameters and their 2- and 3-way interactions explained 77.4% of the MIC variability. After backward elimination a final model remained with 4 predictors, the PSB, the proportion improved, the SD of the latent change, and the correlation between the latent baseline trait and the latent change, explaining 75.6% (adjusted R^2^) (Box S3.2).

**Box S3.2** Regression model for MIC_APM_, model B

Coefficients:

Estimate Std. Error t value Pr(>|t|)

(Intercept) 0.871043 0.002170 401.40 <2e-16 ***

prop.imp.par -0.179037 0.003901 -45.89 <2e-16 ***

psb.par -1.378283 0.018366 -75.04 <2e-16 ***

psb.par:sd.tetchs.par 0.649884 0.017695 36.73 <2e-16 ***

psb.par:cor.t1.ch.par -0.465321 0.010617 -43.83 <2e-16 ***

prop.imp.par:psb.par 2.825501 0.033015 85.58 <2e-16 ***

prop.imp.par:psb.par:sd.tetchs.par -1.364594 0.031810 -42.90 <2e-16 ***

prop.imp.par:psb.par:cor.t1.ch.par 0.885814 0.019086 46.41 <2e-16 ***

---

Signif. codes: 0 ‘***’ 0.001 ‘**’ 0.01 ‘*’ 0.05 ‘.’ 0.1 ‘ ’ 1

Multiple R-squared: 0.7562, Adjusted R-squared: 0.7561

F-statistic: 6827 on 7 and 15403 DF, p-value: < 2.2e-16

The most important predictors were the present state bias and the proportion improved, together explaining 69.3% of the variability in the MIC estimates. Fig. S3.2 shows how MIC_APM_ (model B) was biased by the PSB in combination with the proportion improved. Again, we have seen this effect in an earlier study.^[[6]](#footnote-6)^


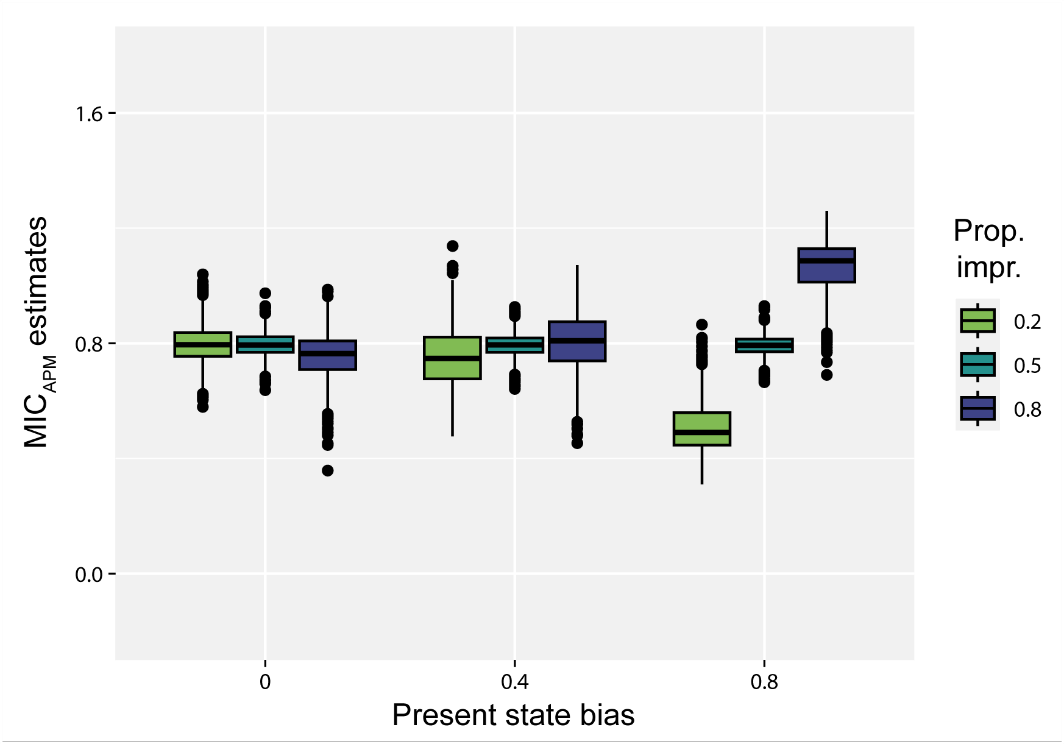


**Fig. S3.2** Distribution of MIC_APM_ (model B) estimates by the present state bias and the proportion improved. The true MIC was 0.8.

## 3.3. Adjusted predictive modeling MIC, model C

All simulation parameters and their 2- and 3-way interactions explained 77.7% of the MIC variability. After backward elimination a final model remained with 3 predictors, the PSB, the proportion improved, and the correlation between the latent baseline trait and the latent change, explaining 74.9% (adjusted R^2^) (Box S3.3).

The most important predictors were – again – the present state bias and the proportion improved, together explaining 69.0% of the variability in the MIC estimates. Fig. S3.3 shows how MIC_APM_ (model C) was biased by the PSB in combination with the proportion improved. Again, we have seen this effect in an earlier study.^[[7]](#footnote-7)^

**Box S3.3** Regression model for MIC_APM_, model C

Coefficients:

Estimate Std. Error t value Pr(>|t|)

(Intercept) 0.859187 0.002235 384.38 <2e-16 ***

prop.imp.par -0.144229 0.004031 -35.78 <2e-16 ***

psb.par -0.729026 0.005108 -142.73 <2e-16 ***

psb.par:cor.t1.ch.par -0.487316 0.010924 -44.61 <2e-16 ***

prop.imp.par:psb.par 1.458364 0.009190 158.69 <2e-16 ***

prop.imp.par:psb.par:cor.t1.ch.par 0.942254 0.019652 47.95 <2e-16 ***

---

Signif. codes: 0 ‘***’ 0.001 ‘**’ 0.01 ‘*’ 0.05 ‘.’ 0.1 ‘ ’ 1

Multiple R-squared: 0.7491, Adjusted R-squared: 0.749

F-statistic: 9138 on 5 and 15306 DF, p-value: < 2.2e-16


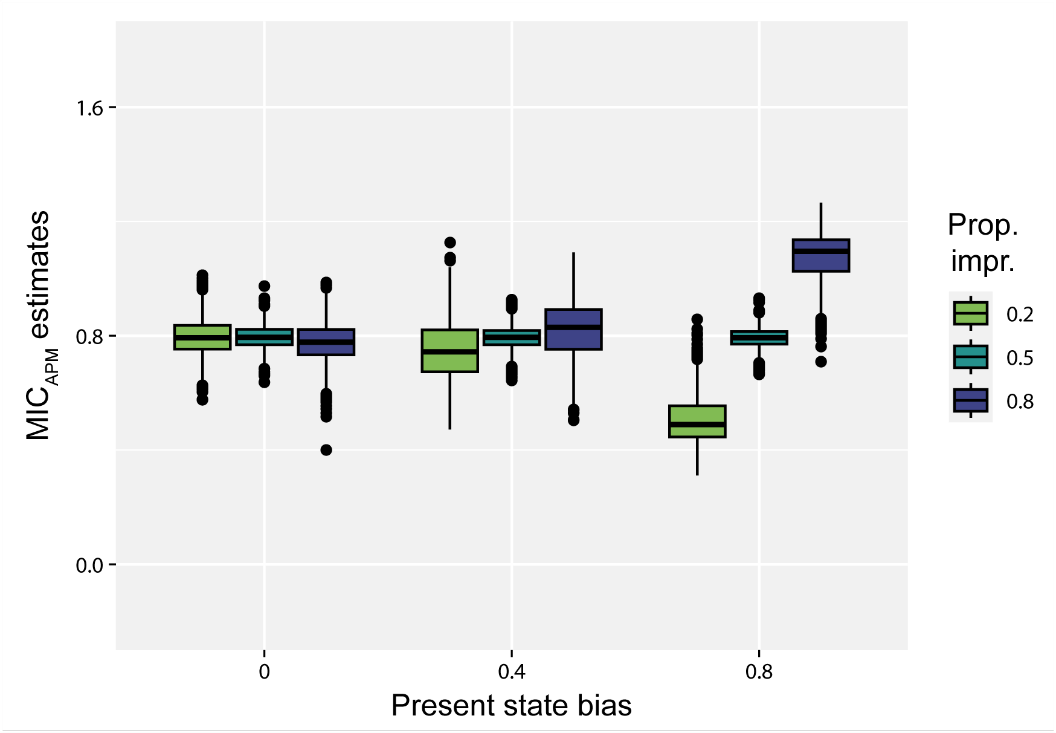


**Fig. S3.3** Distribution of MIC_APM_ (model C) estimates by the present state bias and the proportion improved. The true MIC was 0.8.

## 3.4. LCFA-based MIC, model C

All simulation parameters and their 2- and 3-way interactions explained only 18.1% of the MIC variability. After backward elimination a final model remained with a single predictor, the proportion improved, explaining 12.5% (adjusted R^2^) (Box S3.4). Fig. S3.4 shows how MIC_LCFA_ (model C) was biased by the proportion improved. The effect was minimal.

**Box S3.4** Regression model for MIC_LCFA_, model C

Coefficients:

Estimate Std. Error t value Pr(>|t|)

(Intercept) 0.854305 0.001228 695.9 <2e-16 ***

prop.imp.par -0.103248 0.002211 -46.7 <2e-16 ***

---

Signif. codes: 0 ‘***’ 0.001 ‘**’ 0.01 ‘*’ 0.05 ‘.’ 0.1 ‘ ’ 1

Multiple R-squared: 0.1247, Adjusted R-squared: 0.1246

F-statistic: 2181 on 1 and 15310 DF, p-value: < 2.2e-16


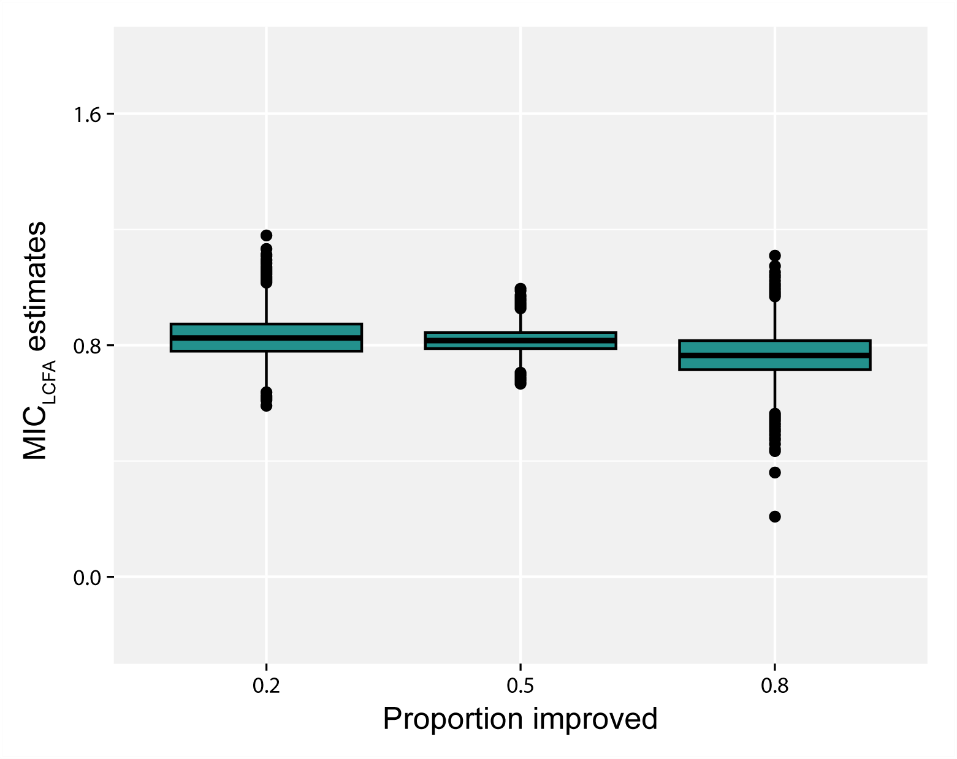


**Fig. S3.4** Distribution of $\mathrm{MIC}_{\mathrm{LCFA}}$ (model C) estimates by the proportion improved. The true MIC was 0.8.

# 4. Recovering the transition ratings reliability

Across the simulated datasets, we estimated the reliability of the transition ratings (TRs) using the various LCFA-models (A-C). Because the TRs reliability was simulated to be 0.4 or 0.7, we analyzed the TRs reliability *residuals* (i.e., the estimated TRs reliability minus the true TRs reliability). Positive residuals mean over-estimation of the TRs reliability and negative residuals mean under-estimation of the TRs reliability. Fig. 4.1 shows that the TRs reliability was nicely recovered when using models A-C, although models B and C showed a small mean under-estimation, but a slightly higher precision than model A. The precision appeared to be impacted by the strength of $\theta^{B}$, the construct component that was targeted by the SIM but not by the AUX.


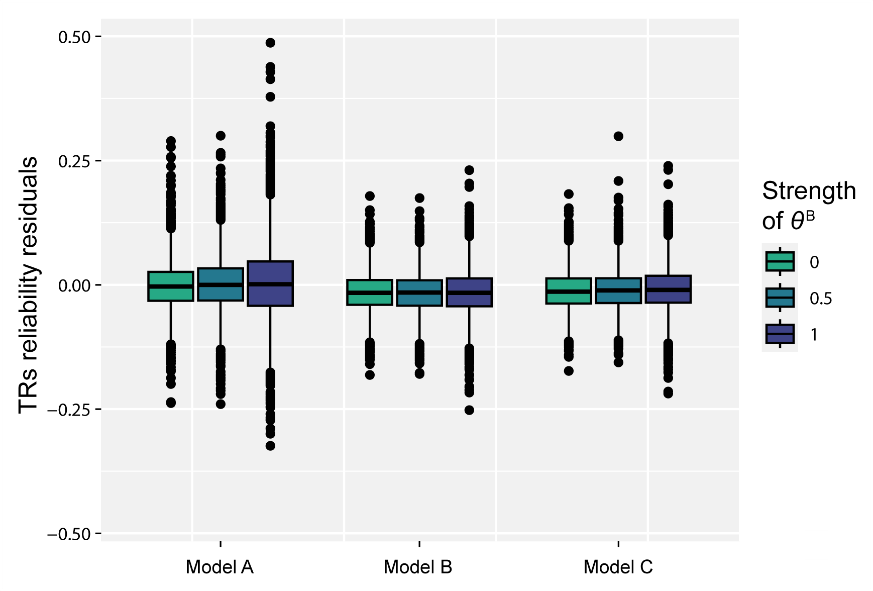


**Fig. S4.1** Distribution of the TRs reliability residual (estimated TRs reliability minus true TRs reliability) by LCFA-model and strength of component $\theta^{B}$

# 5. Old adjusted predictive modeling MIC

For comparison with the improved APM-method, across the simulated datasets, we calculated the MIC according to the “old” APM-method,^[[8]](#footnote-8)^ the best method that does not require fitting an LCFA-model. Table S5.1 shows the results in comparison with the “improved” APM-method,^[[9]](#footnote-9)^ using the various LCFA-models. The old APM-method showed no bias but less precision than the improved APM-method, as demonstrated by the difference in RMSE-values.

**Table S5.1** Performance of the methods to estimate the MIC by estimation method (true MIC was 0.8). Values between brackets are Monte Carlo standard errors

| **Performance measure** | **Old APM-method** | **Improved APM-method** | | |
| --- | --- | --- | --- | --- |
|  |  | **Model A** | **Model B** | **Model C** |
| N | 15,552 | 15,131 | 15,411 | 15,312 |
| Mean estimate | 0.792 | 0.796 | 0.785 | 0.789 |
| Bias | -0.008 (0.003) | -0.004 (0.001) | -0.016 (0.001) | -0.011 (0.001) |
| RMSE | 0.349 (0.032) | 0.168 (0.019) | 0.152 (0.018) | 0.154 (0.018) |

N, number of datasets/converged models; SE, standard error; RMSE, root mean squared error;

APM, adjusted predictive modeling method; A-C, LCFA-model used

Furthermore, we regressed the MIC estimates from the old APM-method on the simulation parameters. All simulation parameters and their 2- and 3-way interactions explained 98.7% of the MIC variability. After backward elimination a final model remained with 3 predictors, the proportion improved, the SD of the latent change, and the reliability of the TRs, explaining 95.7% (adjusted R^2^) (Box S5.1).

**Box S5.1** Regression model for $\mathrm{MIC}_{\mathrm{APM}}^{\mathrm{old}}$

Coefficients:

Estimate Std. Error t value Pr(>|t|)

(Intercept) 0.241632 0.006093 39.66 <2e-16 ***

sd.tetchs.par -0.684984 0.004063 -168.57 <2e-16 ***

rel.trt.par 1.028103 0.008461 121.52 <2e-16 ***

sd.tetchs.par:prop.imp.par 2.466329 0.008594 286.98 <2e-16 ***

sd.tetchs.par:rel.trt.par:prop.imp.par -2.048695 0.015028 -136.33 <2e-16 ***

---

Signif. codes: 0 ‘***’ 0.001 ‘**’ 0.01 ‘*’ 0.05 ‘.’ 0.1 ‘ ’ 1

Multiple R-squared: 0.9565, Adjusted R-squared: 0.9565

F-statistic: 8.552e+04 on 4 and 15547 DF, p-value: < 2.2e-16

The most important predictors were the proportion improved and the SD of the latent change, together explaining 90.5% of the MIC variability. Fig. S5.1 shows how $\mathrm{MIC}_{\mathrm{APM}}^{\mathrm{old}}$ was still biased (despite the adjustment) by the proportion improved, and the SD of the latent change.


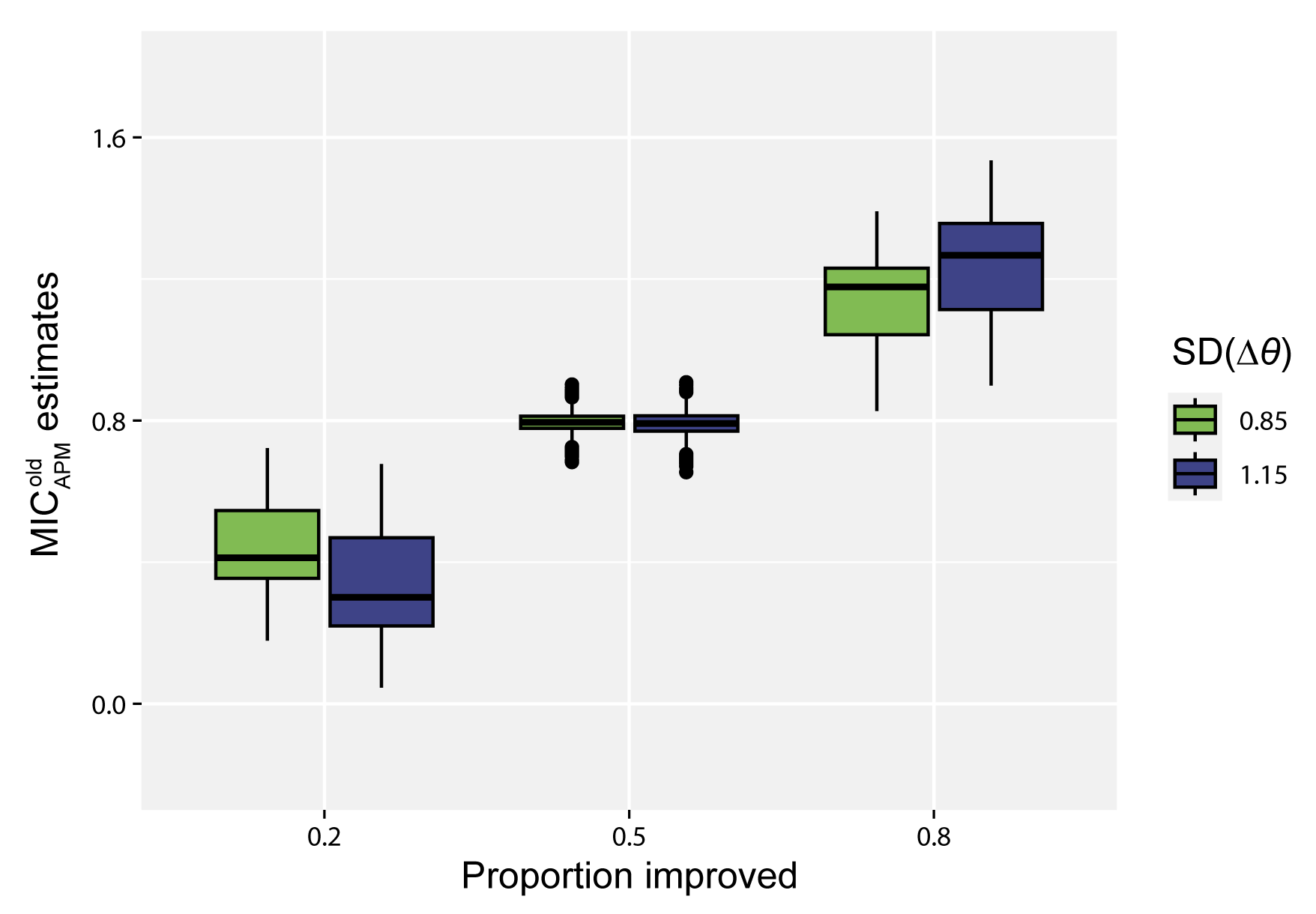


**Fig. S5.1** Distribution of $\mathrm{MIC}_{\mathrm{APM}}^{\mathrm{old}}$ estimates by the proportion improved and the standard deviation of the latent change ($\Delta\theta$). The true MIC was 0.8.

# 6. Alternative true MIC values

On request of one of the reviewers, we investigated the robustness of recovering the true MIC, by repeating the simulations and analyses using two alternative values for the true MIC, namely 0.5 and 1.0.^[[10]](#footnote-10)^ We varied a more limited number of parameters (Table 6.1), and simulated 2 x 648 different datasets. The MIC was estimated using both the APM-method and the LCFA-method, using model C as the LCFA-model to estimate the TRs reliability and the latent MIC. Figure 6.1 shows that the true MIC was accurately recovered, as accurate and precise as when the true MIC was 0.8 (compare with Fig. 3 in the article).

**Table 6.1** Parameters (not) varying across 1296 simulated datasets

| **Parameter** | **Values** | **Explanation** |
| --- | --- | --- |
| Mean latent trait $\theta$ at T1 ($\overline{\theta_{T1}}$) | 0 | $\overline{\theta_{T1}}$ reflects the average (severity) level of the latent trait at T1 |
| SD latent change (SD($\Delta\theta$)) | 1 | SD($\Delta\theta$) reflects the variability in the latent change ($\Delta\theta$) between T1 and T2 |
| Correlation between $\theta_{T1}$ and $\Delta\theta$ | -0.50, 0.00 | Values are correlation coefficients |
| Reliability of SIM_T1_ | 0.50, 0.80 | Values are reliability coefficients |
| Reliability of the TRs | 0.40, 0.70 | Values are reliability coefficients |
| Present state bias (PSB) | 0, 0.4, 0.8 | Proportion of $\theta_{T2}-\overline{\theta_{T1}}$ that is included in the weighted latent change (${\Delta\theta}_{w}$) |
| Proportion improved | 0.2, 0.5, 0.8 | Proportion of patients having a true latent change ($\Delta\theta$) greater than their individual MIC |
| Relative strength of the unique AUX factor ($\xi$) | 0, 0.2, 0.5 | Values are ratios of the mean slope parameter of $\xi$ to the mean slope parameter of $\theta^{A}$ |
| Relative strength of $\theta^{B}$ | 0, 0.5, 1 | Ratio of the SD of $\theta^{B}$ to the SD of $\theta^{A}$ |
| Correlation between $\xi_{T1}$ and $\xi_{T2}$ | 0.50 | Values are correlation coefficients |
| Correlation between $\eta_{T1}$ and $\eta_{T2}$ | 0.50 | Values are correlation coefficients |

SD, Standard deviation; T1, Baseline; T2, Follow-up; SIM, single-item measure; TRs, transition ratings; AUX, auxiliary patient-reported outcome measure


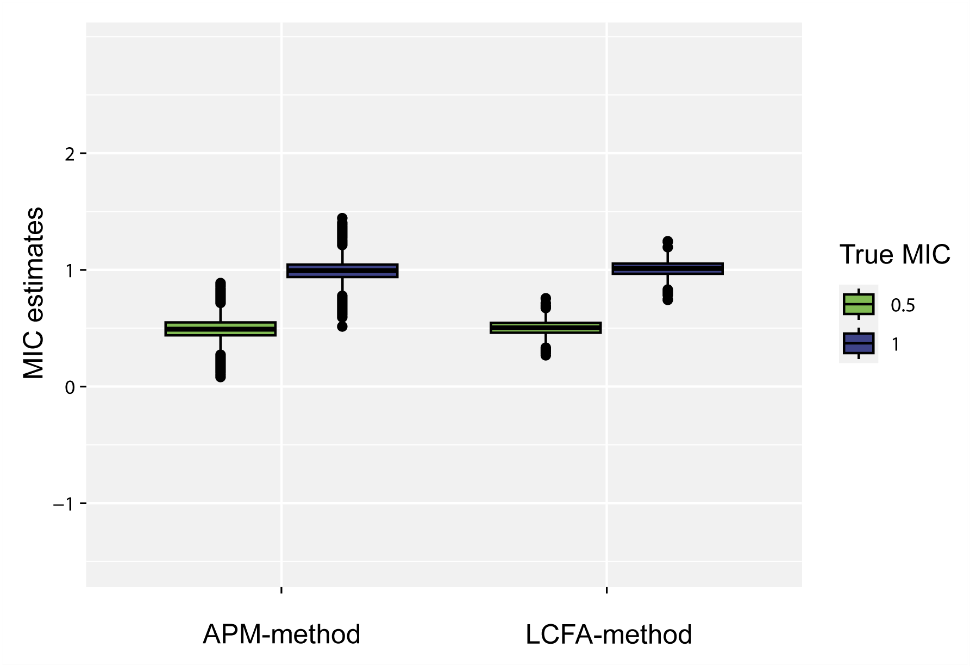


**Fig. 6.1** Distribution of MIC estimates by method and true MIC

Note that 1 unit in the metric of the latent construct represents, by default, 1 standard deviation (SD) of the latent baseline distribution. An MIC of 1 in the same metric thus means that, on average, patients start to value their change as important if that change exceeds 1 baseline SD. 0.5 or 1 SD is sometimes used as benchmark for a “distribution-based” MIC.^[[11]](#footnote-11)^ MICs in the range of 0.5 – 1 in the metric of the latent construct, are probably realistic. In our real data example, we estimated a latent MIC of 1 – 1.2.

# 7. R-code for simulation and estimation

# This code simulates 2 datasets (baseline, T1; follow-up, T2) for a sample

# of N persons, creating the scores of a single item measure (SIM) and

# the item scores of a 10 item PROM, and the responses to a global transition

# question. The SIM is simulated as a numeric rating scale (NRS).

# Theta is the underlying trait of the SIM and the transition ratings (TR).

# Theta consists of 2 components theta-A and theta-B;

# theta-A is underlying the multi-item PROM scores.

rm(list=ls(all=TRUE)) # remove all objects

# Acquire packages

library(psych)

library(ggplot2)

library(mirt)

library(lavaan)

library(MASS)

library(e1071)

library(semTools)

library(haven)

library(dplyr)

library(beepr)

## SET THE PARAMETERS FOR THE SIMULATIONS

nr=1 # set number of times each combination must be repeated

## Create vectors for parameters to vary across the simulated samples

par.sample.size <- c(2000)

# parameter controlling the sample size

par.mn.tet1s <- c(-1, 0, 1) # mean theta T1 (k2)

# mean theta score at T1, creates skewed observed scores

par.sd.tet1s <- c(1) # SD theta T1

# parameter controlling the SD of theta T1;

par.mn.imic <- c(0.8) # mean iMIC

# parameter controlling the mean iMIC (= genuine MIC; gMIC)

par.sd.imic <- c(0.075) # SD iMIC

# parameter controlling the SD of the iMICs

# TAKE CARE: some iMIC values should not approach zero as this

# will not be realistic for a 'minimal important' improvement

par.sd.tetchs <- c(0.85, 1.15) # SD theta change (k6)

# parameter controlling the SD of theta change (T2-T1);

par.cor.t1.ch <- c(-0.5, 0) # cor between theta T1 and change (k7)

# parameter controlling the correlation between theta T1 the theta change;

# values represent correlation coefficients

par.cor.t1.imic <- c(0) # cor between theta T1 and iMIC

# parameter controlling the correlation between theta T1 and the iMICs;

# value represents a correlation coefficient

par.prop.imp <- c(0.2, 0.5, 0.8) # 'true' proportion improved (k9)

# parameter controlling the 'true' proportion improved, that is

# the proportion patients whose latent change exceed their iMIC.

par.rel.trs <- c(0.4, 0.7) # reliability of transition ratings (k8)

# parameter controlling the reliability of the transition ratings (TRs).

# Actually, the parameter controls the reliability of the "perceived change"

# relative to the weighted change.

par.psb <- c(0, 0.4, 0.8) # PSB (k11)

# parameter controlling the present state bias of the transition

# ratings (TRs); values represent the average proportion of present state

# that is included in the "weighted change"

par.rel.sim <- c(0.5, 0.8) # reliability of the SIM (k3)

# parameter controlling the reliability of the SIM (at T1)

par.strength.Xi <- c(0, 0.2, 0.5) # strength of unique AUX factor (k5)

# parameter controls the strength of the unique AUX factor underlying the

# AUX items, relative to the common factor underlying the SIMs (theta-A).

par.str.compB <- c(0, 0.5, 1) # Strength of theta component B relative to A (k10)

# parameter controlling the relative strength of theta component B,

# values; 1 means that the SD of component B is the same as

# the SD of component A.

par.cor.Xi1.Xi2 <- c(0.5, 0.9) # Correlation between the unique AUX factors (k4)

# over time; correlation = 1 means that the unique AUX factor does not change.

par.cor.Et1.Et2 <- c(0.5, 0.9) # Correlation between the unique SIM factors (k1)

# over time; correlation = 1 means that the unique SIM factor does not change.

## Calculate number of combinations and total number of simulations

npc <- length(par.sample.size) * length(par.mn.tet1s) * length(par.sd.tet1s) *

length(par.mn.imic) * length(par.sd.imic) * length(par.sd.tetchs) *

length(par.cor.t1.ch) * length(par.cor.t1.imic) * length(par.prop.imp) *

length(par.rel.trs) * length(par.psb) * length(par.rel.sim) *

length(par.strength.Xi) * length(par.str.compB) *

length(par.cor.Xi1.Xi2) * length(par.cor.Et1.Et2)

npc # total number of combinations

nps <- npc * nr

nps # total number of simulated samples

index <- 0

## Create starting point for dataframe "dfp" to hold results of the simulations

sample.size.par <- as.numeric(rep(NA, nps))

mn.tet1s.par <- as.numeric(rep(NA, nps))

dfp <- data.frame(sample.size.par, mn.tet1s.par) # creates dataframe "dfp"

## create the other variables in "dfp"

dfp$sd.tet1s.par <- as.numeric(rep(NA, nps))

dfp$mn.imic.par <- as.numeric(rep(NA, nps))

dfp$sd.imic.par <- as.numeric(rep(NA, nps))

dfp$sd.tetchs.par <- as.numeric(rep(NA, nps))

dfp$cor.t1.ch.par <- as.numeric(rep(NA, nps))

dfp$cor.t1.imic.par <- as.numeric(rep(NA, nps))

dfp$prop.imp.par <- as.numeric(rep(NA, nps))

dfp$rel.trs.par <- as.numeric(rep(NA, nps))

dfp$psb.par <- as.numeric(rep(NA, nps))

dfp$rel.sim.par <- as.numeric(rep(NA, nps))

dfp$strength.Xi.par <- as.numeric(rep(NA, nps))

dfp$str.compB.par <- as.numeric(rep(NA, nps))

dfp$cor.Xi1.Xi2.par <- as.numeric(rep(NA, nps))

dfp$cor.Et1.Et2.par <- as.numeric(rep(NA, nps))

dfp$prop.imp.trs <- as.numeric(rep(NA, nps)) # prop. improved based on TRs

dfp$prop.imp.tru <- as.numeric(rep(NA, nps)) # prop. imp. based on true change

dfp$mn.sim1 <- as.numeric(rep(NA, nps))

dfp$sd.sim1 <- as.numeric(rep(NA, nps))

dfp$skew.sim1 <- as.numeric(rep(NA, nps))

dfp$kurt.sim1 <- as.numeric(rep(NA, nps))

dfp$flor.sim1 <- as.numeric(rep(NA, nps))

dfp$ceil.sim1 <- as.numeric(rep(NA, nps))

dfp$mn.sim2 <- as.numeric(rep(NA, nps))

dfp$sd.sim2 <- as.numeric(rep(NA, nps))

dfp$skew.sim2 <- as.numeric(rep(NA, nps))

dfp$kurt.sim2 <- as.numeric(rep(NA, nps))

dfp$flor.sim2 <- as.numeric(rep(NA, nps))

dfp$ceil.sim2 <- as.numeric(rep(NA, nps))

dfp$mn.simdif <- as.numeric(rep(NA, nps))

dfp$sd.simdif <- as.numeric(rep(NA, nps))

dfp$skew.simdif <- as.numeric(rep(NA, nps))

dfp$kurt.simdif <- as.numeric(rep(NA, nps))

dfp$flor.simdif <- as.numeric(rep(NA, nps))

dfp$ceil.simdif <- as.numeric(rep(NA, nps))

dfp$mn.xo1 <- as.numeric(rep(NA, nps)) # mean final AUX score at T1

dfp$sd.xo1 <- as.numeric(rep(NA, nps)) # SD final AUX score at T1

dfp$skew.xo1 <- as.numeric(rep(NA, nps))

dfp$kurt.xo1 <- as.numeric(rep(NA, nps))

dfp$flor.xo1 <- as.numeric(rep(NA, nps))

dfp$ceil.xo1 <- as.numeric(rep(NA, nps))

dfp$mn.xo2 <- as.numeric(rep(NA, nps)) # mean final AUX score at T2

dfp$sd.xo2 <- as.numeric(rep(NA, nps)) # SD final AUX score at T2

dfp$skew.xo2 <- as.numeric(rep(NA, nps))

dfp$kurt.xo2 <- as.numeric(rep(NA, nps))

dfp$flor.xo2 <- as.numeric(rep(NA, nps))

dfp$ceil.xo2 <- as.numeric(rep(NA, nps))

dfp$mn.xoc <- as.numeric(rep(NA, nps)) # mean change score all subjects

dfp$sd.xoc <- as.numeric(rep(NA, nps)) # SD change score of all subjects

dfp$skew.xoc <- as.numeric(rep(NA, nps))

dfp$kurt.xoc <- as.numeric(rep(NA, nps))

dfp$flor.xoc <- as.numeric(rep(NA, nps))

dfp$ceil.xoc <- as.numeric(rep(NA, nps))

dfp$rel.xo1 <- as.numeric(rep(NA, nps)) # reliability observed score T1

dfp$mn.imic <- as.numeric(rep(NA, nps)) # mean iMIC (= gMIC)

dfp$sd.imic <- as.numeric(rep(NA, nps)) # SD of iMIC

dfp$cor.simdif.trs <- as.numeric(rep(NA, nps)) # correlation between TRs and observed SIM change score

dfp$pcor.simdif.trs <- as.numeric(rep(NA, nps)) # BISERIAL correlation between TRs and observed SIM change score

dfp$cor.sim1.xo1 <- as.numeric(rep(NA, nps))

dfp$cor.sim2.xo2 <- as.numeric(rep(NA, nps))

dfp$rel.trs.ma <- as.numeric(rep(NA, nps)) # Reliability TRs model A

dfp$rel.trs.mb <- as.numeric(rep(NA, nps)) # Reliability TRs model B

dfp$rel.trs.mc <- as.numeric(rep(NA, nps)) # Reliability TRs model C

dfp$psb.mc <- as.numeric(rep(NA, nps)) # PSB estimated model C

dfp$mic.pred <- as.numeric(rep(NA, nps)) # MIC predicted

dfp$mic.adj.old <- as.numeric(rep(NA, nps)) # MIC adjusted old (2017)

dfp$mic.adj.ma <- as.numeric(rep(NA, nps)) # MIC adjusted 2022 model A

dfp$mic.adj.mb <- as.numeric(rep(NA, nps)) # MIC adjusted 2022 model B

dfp$mic.adj.mc <- as.numeric(rep(NA, nps)) # MIC adjusted 2022 model C

dfp$mic.theta.mc <- as.numeric(rep(NA, nps)) # latent MIC based model C

dfp$rel.sim1.mc <- as.numeric(rep(NA, nps)) # Reliability of SIM1 (model C)

dfp$mic.lcfa.mc <- as.numeric(rep(NA, nps)) # MIC based on true SD model C

dfp$fit.cfi.ma <- as.numeric(rep(NA, nps)) # CFI of model A

dfp$fit.tli.ma <- as.numeric(rep(NA, nps)) # TLI

dfp$fit.rms.ma <- as.numeric(rep(NA, nps)) # RMSEA

dfp$fit.srmr.ma <- as.numeric(rep(NA, nps)) # SRMR

dfp$fit.cfi.mb <- as.numeric(rep(NA, nps)) # CFI of model B

dfp$fit.tli.mb <- as.numeric(rep(NA, nps)) # TLI

dfp$fit.rms.mb <- as.numeric(rep(NA, nps)) # RMSEA

dfp$fit.srmr.mb <- as.numeric(rep(NA, nps)) # SRMR

dfp$fit.cfi.mc <- as.numeric(rep(NA, nps)) # CFI of model C

dfp$fit.tli.mc <- as.numeric(rep(NA, nps)) # TLI

dfp$fit.rms.mc <- as.numeric(rep(NA, nps)) # RMSEA

dfp$fit.srmr.mc <- as.numeric(rep(NA, nps)) # SRMR

dfp$mn.tet1s.sim <- as.numeric(rep(NA, nps)) # mean latent theta T1 as simulated

dfp$sd.tet1s.sim <- as.numeric(rep(NA, nps)) # mean latent theta T1 as simulated

dfp$mn.tetchs.sim <- as.numeric(rep(NA, nps)) # mean latent change as simulated

dfp$sd.tetchs.sim <- as.numeric(rep(NA, nps)) # SD latent change as simulated

dfp$mn.change.mc <- as.numeric(rep(NA, nps)) # mean latent change model C

dfp$sd.change.mc <- as.numeric(rep(NA, nps)) # mean latent change model C

dfp$item.x <- as.numeric(rep(NA, nps)) # random item chosen for model A

## Create variable to hold the random numbers

Randomseed <- matrix(rep(NA, nps*626), ncol=626)

### SIMULATE A SET OF ITEM PARAMETERS

# The b2 parameters (the 'middle' location parameters) consist of a series

# of numbers between -0.8 and +0.8 with intervals of 0.4. The b1 parameters

# (the first location parameters) are based on b2 minus 1 plus a random

# distribution of b2/4. The b3 parameters (the third location parameters)

# are based on b2 plus 1 plus a different random distribution of b2/4.

# The a parameters consist of 1.7 + b2/2, randomly distributed.

set.seed(12345)

b2 <- c(-0.8, -0.8, -0.4, -0.4, 0, 0, 0.4, 0.4, 0.8, 0.8)

bc <- b2/4

b1 <- b2 - 1 + sample(bc)

b3 <- b2 + 1 + sample(bc)

a1 <- sample( 1.7+b2/2 )

cf.simb <- as.matrix( data.frame(a1,b1,b2,b3) )

round(cf.simb, 3)

round(colMeans(cf.simb), 3)

cf.simb <- as.data.frame(cf.simb)

# Transform b-parameters to d-parameters ('mirt' works with d-parameters)

# difficulty (b) = easiness (d) / -a

cf.sim <- cf.simb

colnames(cf.sim) <- c("a1","d1","d2","d3")

cf.sim$d1 <- -cf.simb$b1*cf.sim$a1

cf.sim$d2 <- -cf.simb$b2*cf.sim$a1

cf.sim$d3 <- -cf.simb$b3*cf.sim$a1

# round(cf.sim, 3)

# Simulate dataset using 'mirt'

a1 <- as.matrix(cf.sim[ , 1])

d1 <- as.matrix(cf.sim[ , -1])

# Create a dataset and check reliability

# Create dataset with theta = N(0,1)

set.seed(12345)

theta.sim <- as.matrix( rnorm(20000, 0, 1) )

dat <- simdata(a1, d1, 20000, itemtype="graded", Theta=theta.sim)

dat <- as.data.frame(dat)

round( psych::alpha(dat)$total$raw_alpha, 3 ) # Cronbach's alpha

#############################################################################

### SIMULATIONS

start.time <- Sys.time()

# set.seed(10)

## --------------------------------

## Create repetitions and perform the analyses

for(k1 in 1:length(par.cor.Et1.Et2)) {

for(k2 in 1:length(par.mn.tet1s)) {

for(k3 in 1:length(par.rel.sim)) {

for(k4 in 1:length(par.cor.Xi1.Xi2)) {

for(k5 in 1:length(par.strength.Xi)) {

for(k6 in 1:length(par.sd.tetchs)) {

for(k7 in 1:length(par.cor.t1.ch)) {

for(k8 in 1:length(par.rel.trs)) {

for(k9 in 1:length(par.prop.imp)) {

for(k10 in 1:length(par.str.compB)) {

for(k11 in 1:length(par.psb)) {

for(k12 in 1:nr) {

index <- (k1-1)*length(par.mn.tet1s)*length(par.rel.sim)*

length(par.cor.Xi1.Xi2)*length(par.strength.Xi)*length(par.sd.tetchs)*

length(par.cor.t1.ch)*length(par.rel.trs)*length(par.prop.imp)*

length(par.str.compB)*length(par.psb)*nr +

(k2-1)*length(par.rel.sim)*

length(par.cor.Xi1.Xi2)*length(par.strength.Xi)*length(par.sd.tetchs)*

length(par.cor.t1.ch)*length(par.rel.trs)*length(par.prop.imp)*

length(par.str.compB)*length(par.psb)*nr +

(k3-1)*length(par.cor.Xi1.Xi2)*length(par.strength.Xi)*length(par.sd.tetchs)*

length(par.cor.t1.ch)*length(par.rel.trs)*length(par.prop.imp)*

length(par.str.compB)*length(par.psb)*nr +

(k4-1)*length(par.strength.Xi)*length(par.sd.tetchs)*

length(par.cor.t1.ch)*length(par.rel.trs)*length(par.prop.imp)*

length(par.str.compB)*length(par.psb)*nr +

(k5-1)*length(par.sd.tetchs)*

length(par.cor.t1.ch)*length(par.rel.trs)*length(par.prop.imp)*

length(par.str.compB)*length(par.psb)*nr +

(k6-1)*length(par.cor.t1.ch)*length(par.rel.trs)*length(par.prop.imp)*

length(par.str.compB)*length(par.psb)*nr +

(k7-1)*length(par.rel.trs)*length(par.prop.imp)*

length(par.str.compB)*length(par.psb)*nr +

(k8-1)*length(par.prop.imp)*

length(par.str.compB)*length(par.psb)*nr +

(k9-1)*length(par.str.compB)*length(par.psb)*nr +

(k10-1)*length(par.psb)*nr +

(k11-1)*nr + k12

print(index)

Randomseed[index,] <- .Random.seed

###

( dfp$sample.size.par[index] <- N <- par.sample.size )

( dfp$mn.tet1s.par[index] <- par.mn.tet1s[k2] )

( dfp$sd.tet1s.par[index] <- par.sd.tet1s )

( dfp$mn.imic.par[index] <- par.mn.imic )

( dfp$sd.imic.par[index] <- par.sd.imic )

( dfp$sd.tetchs.par[index] <- par.sd.tetchs[k6] )

( dfp$cor.t1.ch.par[index] <- par.cor.t1.ch[k7] )

( dfp$cor.t1.imic.par[index] <- par.cor.t1.imic )

( dfp$prop.imp.par[index] <- par.prop.imp[k9] )

( dfp$rel.trs.par[index] <- par.rel.trs[k8] )

( dfp$psb.par[index] <- par.psb[k11] )

( dfp$rel.sim.par[index] <- par.rel.sim[k3] )

( dfp$strength.Xi.par[index] <- par.strength.Xi[k5] )

( dfp$str.compB.par[index] <- par.str.compB[k10] )

( dfp$cor.Xi1.Xi2.par[index] <- par.cor.Xi1.Xi2[k4] )

( dfp$cor.Et1.Et2.par[index] <- par.cor.Et1.Et2[k1] )

## Simulate theta as composed of 2 components (A and B)

## Where the AUX items load on only 1 component (A)

## and the SIM and TRs load on both.

### Create theta T1 (baseline latent trait underlying the SIM)

( N = par.sample.size )

( R1 <- par.cor.t1.ch[k7] ) # correlation between tet1s and tetchs

( R2 <- par.cor.Et1.Et2[k1] ) # correlation between Eta_T1 and Eta_T2

( R3 <- par.cor.Xi1.Xi2[k4] ) # correlation between Xi_T1 and Xi_T2

## Create 2 partitions for theta (A and B)

Sigma <- matrix(rep(0,64),8,8)

diag(Sigma) <- 1

Sigma[1,3] <- R1

Sigma[2,4] <- R1

Sigma[3,1] <- R1

Sigma[4,2] <- R1

Sigma[5,6] <- R2

Sigma[6,5] <- R2

Sigma[7,8] <- R3

Sigma[8,7] <- R3

Sigma

tets <- mvrnorm(N, rep(0, 8), Sigma)

mean(tets[,1])

mean(tets[,2])

sd(tets[,1])

sd(tets[,2])

cor(tets[,1],tets[,3]) # R1

cor(tets[,2],tets[,4]) # R1

cor(tets[,5],tets[,6]) # R2

cor(tets[,7],tets[,8]) # R3

class(tets)

# Component A of theta T1

par.str.compB[k10]

par.mn.tet1s[k2]

tet1a <- tets[,1]*par.sd.tet1s/sd(tets[,1]) # adjust SD

# Component B of theta T1

tet1b <- tets[,2]*par.str.compB[k10]*par.sd.tet1s/sd(tets[,2]) # adjust SD

# cor(tet1a,tet1b)

# Transform tet1s components such that tet1s (tet1a+tet1b) variance=1 has

tet1s <- tet1a + tet1b

var(tet1s)

red.factor <- sd(tet1s) # reduction factor

tet1a <- tet1a / red.factor # tet1a rescaled

tet1b <- tet1b / red.factor # tet1b rescaled

mean(tet1a)

sd(tet1a)

mean(tet1b)

sd(tet1b)

# Adjust means

par.mn.tet1s[k2]

tet1a <- tet1a-mean(tet1a)+(1-0.5*par.str.compB[k10])*par.mn.tet1s[k2] # adjust mean

tet1b <- tet1b-mean(tet1b)+0.5*par.str.compB[k10]*par.mn.tet1s[k2] # adjust mean

mean(tet1a)

sd(tet1a)

mean(tet1b)

sd(tet1b)

tet1s <- tet1a + tet1b

( dfp$mn.tet1s.sim[index] <- mean(tet1s) )

( dfp$sd.tet1s.sim[index] <- sd(tet1s) )

### Create theta change

# Component A of theta change

tetca <- tets[,3]

cor(tet1a,tetca)

tetca <- tetca - mean(tetca) # make mean = 0

tetca <- (tetca/sd(tetca))*par.sd.tetchs[k6] # transform SD of tetchs

mean(tetca)

sd(tetca)

# Component B of theta change

tetcb <- tets[,4]

# cor(tet1b,tetcb)

tetcb <- tetcb - mean(tetcb) # make mean = 0

tetcb <- (tetcb/sd(tetcb))*par.sd.tetchs[k6]*par.str.compB[k10] # transform SD of tetchs

mean(tetcb)

sd(tetcb)

tetchs <- tetca + tetcb

sd(tetchs)

( red.factor <- sd(tetchs)/par.sd.tetchs[k6] ) # reduction factor

tetca <- tetca / red.factor

tetcb <- tetcb / red.factor

tetchs <- tetca + tetcb # tetchs redefined/rescaled

sd(tetchs)

mean(tetchs)

( qtl <- quantile(tetchs, prob=(1-par.prop.imp[k9])) )

( mean.tetch <- par.mn.imic - qtl ) # Estimate mean theta change to get

# the desired proportion improved

tetca <- tetca + mean.tetch # transform mean of tetca

# tetcb <- tetcb + 0 # transform mean of tetcb

# Create tet2a en tet2b

tet2a <- tet1a + tetca

tet2b <- tet1b + tetcb

# Create tet1s, tet2s en tetchs

tetchs <- tetca + tetcb

mean(tetchs)

sd(tetchs)

### Create theta T2 (follow-up)

tet2s <- as.matrix(tet1s + tetchs) # theta T2

tet2ss <- tet2a + tet2b

cor(tet2s,tet2ss)

mean(tet2s)

var(tet2s)

# SD and mean of the true latent change, as simulated

sqrt(var(tet1s)+var(tet2s)-2*cov(tet1s,tet2s))

( dfp$sd.tetchs.sim[index] <- sd(tetchs) )

( dfp$mn.tetchs.sim[index] <- mean(tetchs) )

### Create observed SIM scores

# Note: The latent trait tet1s is assumed to underly the SIM scores and the

# AUX scores. Next to that, both the SIM and the AUX (may) have a unique

# piece of variance (which with respect to measuring tet1s is "error").

# So, we need to simulate "error" for the SIM scores (which determines its

# reliability) that is partly unique SIM variance (Eta1 and Eta2) and partly

# true error. Arbitrarily we choose a 50-50 distribution.

# Add Eta factor and "error"

( rel.sim = par.rel.sim[k3] )

( sd.sim.error <- sqrt(((1-rel.sim)/rel.sim)*sd(tet1s)^2) )

Eta1 <- tets[,5]

Eta2 <- tets[,6]

cor(Eta1,Eta2)

mean(Eta1)

sd(Eta1)

Eta1 <- Eta1*sqrt(sd.sim.error^2/2)

Eta2 <- Eta2*sqrt(sd.sim.error^2/2)

sim1 <- tet1s + Eta1 + rnorm(N, 0, sqrt(sd.sim.error^2/2))

sim2 <- tet2s + Eta2 + rnorm(N, 0, sqrt(sd.sim.error^2/2))

var(tet1s)/var(sim1) # Reliability SIM1 (common variance over total variance)

var(tet2s)/var(sim2) # Reliability SIM2 (common variance over total variance)

# Create integer scores by applying thresholds

# Note that 1 SIM point corresponds to 1 theta SD (this is arbitrary)

SIM1 <- as.numeric(rep(1,N))

SIM1[sim1>-4] <- 2

SIM1[sim1>-3] <- 3

SIM1[sim1>-2] <- 4

SIM1[sim1>-1] <- 5

SIM1[sim1>0] <- 6

SIM1[sim1>1] <- 7

SIM1[sim1>2] <- 8

SIM1[sim1>3] <- 9

SIM1[sim1>4] <- 10

SIM2 <- as.numeric(rep(1,N))

SIM2[sim2>-4] <- 2

SIM2[sim2>-3] <- 3

SIM2[sim2>-2] <- 4

SIM2[sim2>-1] <- 5

SIM2[sim2>0] <- 6

SIM2[sim2>1] <- 7

SIM2[sim2>2] <- 8

SIM2[sim2>3] <- 9

SIM2[sim2>4] <- 10

table(SIM1)

table(SIM2)

cor(sim1,SIM1)

cor(sim2,SIM2)

cor(SIM1,SIM2)

cor(SIM1,tet1s)

cor(SIM2,tet2s)

SIM.dif <- SIM2-SIM1

cor(SIM.dif,(tet2s-tet1s))

( dfp$mn.sim1[index] <- mean(SIM1) )

( dfp$sd.sim1[index] <- sd(SIM1) )

( dfp$skew.sim1[index] <- e1071::skewness(SIM1, type=2) )

( dfp$kurt.sim1[index] <- e1071::kurtosis(SIM2, type=2) )

( dfp$flor.sim1[index] <- table(SIM1)[1] / length(SIM1) )

( dfp$ceil.sim1[index] <- table(SIM1)[length(table(SIM1))] / length(SIM1) )

( dfp$mn.sim2[index] <- mean(SIM2) )

( dfp$sd.sim2[index] <- sd(SIM2) )

( dfp$skew.sim2[index] <- e1071::skewness(SIM2, type=2) )

( dfp$kurt.sim2[index] <- e1071::kurtosis(SIM2, type=2) )

( dfp$flor.sim2[index] <- table(SIM2)[1] / length(SIM2) )

( dfp$ceil.sim2[index] <- table(SIM2)[length(table(SIM2))] / length(SIM2) )

( dfp$mn.simdif[index] <- mean(SIM.dif) )

( dfp$sd.simdif[index] <- sd(SIM.dif) )

( dfp$skew.simdif[index] <- e1071::skewness(SIM.dif, type=2) )

( dfp$kurt.simdif[index] <- e1071::kurtosis(SIM.dif, type=2) )

( dfp$flor.simdif[index] <- table(SIM.dif)[1] / length(SIM.dif) )

( dfp$ceil.simdif[index] <- table(SIM.dif)[length(table(SIM.dif))] / length(SIM.dif) )

# Create the AUX items.

# We assume that the AUX and the SIM are correlated based on a common piece

# of variance - that is the latent trait (construct) underlying the SIM.

# Next to this common variance, we assume that the AUX may measure a different

# construct - that is the unique variance of the AUX (factor Xi).

# Moreover, we assume that the SIM might measure a "broader" construct than

# the AUX.

# Therefore, we simulate the AUX item scores based on 2 factors (component A

# of theta, and factor Xi).

# Create latent traits of unique AUX factor (Xi)

# For simplicity we assume that the correlation between the baseline Xi trait

# and the latent Xi change is the same as between the latent baseline theta trait

# and the latent theta change.

# We create Xi1 with a mean and SD similar to theta T1 component A

Xi1 <- tets[,7]

Xi2 <- tets[,8]

cor(Xi1,Xi2)

# Give Xi1/Xi2 the same variance as tet1a/tet2a

Xi1 <- Xi1 * sd(tet1a)

Xi2 <- Xi2 * sd(tet2a)

mean(tet1a)

sd(tet1a)

mean(Xi1)

sd(Xi1)

mean(tet2a)

sd(tet2a)

mean(Xi2)

sd(Xi2)

### Create AUX items using mirt

### Create 10 AUX items for T1

# By changing the IRT a-parameter we make the unique factor less or more strong

# For instance, if the strength parameter is 0.5, the Xi factor is half as strong

# as the theta factor.

par.strength.Xi[k5]

( a2 <- a1*par.strength.Xi[k5] )

( a2 <- sample(a2,10,replace=F) ) # We resample the values

( a <- cbind(a1,a2) )

### AUX based on theta components A and Xi

aux1 <- cbind(tet1a, Xi1) # 2 factors underlying the AUX T1

head(aux1)

class(aux1)

cor(aux1)

aux2 <- cbind(tet2a, Xi2) # 2 factors underlying the AUX T2

set.seed( sample(10000:20000,1) )

dat1 <- simdata(a, d1, N, itemtype="graded", Theta=aux1)

dat1 <- as.data.frame(dat1)

set.seed( sample(30000:40000,1) )

dat2 <- simdata(a, d1, N, itemtype="graded", Theta=aux2)

dat2 <- as.data.frame(dat2)

xo1 <- rowSums(dat1) # This is the baseline AUX score

( dfp$mn.xo1[index] <- mean(xo1) )

( dfp$sd.xo1[index] <- sd(xo1) )

( dfp$skew.xo1[index] <- skewness(xo1, type=2) )

( dfp$kurt.xo1[index] <- e1071::kurtosis(xo1, type=2) )

( dfp$flor.xo1[index] <- table(xo1)[1] / length(xo1) ) # floor effect

( dfp$ceil.xo1[index] <- table(xo1)[length(table(xo1))] / length(xo1) )# ceiling effect

( dfp$rel.xo1[index] <- psych::alpha(dat1)$total$raw_alpha ) # Cronbach's alpha XO1

xo2 <- rowSums(dat2) # This is the follow-up AUX score

( dfp$mn.xo2[index] <- mean(xo2) )

( dfp$sd.xo2[index] <- sd(xo2) )

( dfp$skew.xo2[index] <- skewness(xo2, type=2) )

( dfp$kurt.xo2[index] <- e1071::kurtosis(xo2, type=2) )

( dfp$flor.xo2[index] <- table(xo2)[1] / length(xo2) )

( dfp$ceil.xo2[index] <- table(xo2)[length(table(xo2))] / length(xo2) )

xoc <- xo2 - xo1 # This is the AUX change score

( dfp$mn.xoc[index] <- mean(xoc) )

( dfp$sd.xoc[index] <- sd(xoc) )

( dfp$skew.xoc[index] <- skewness(xoc, type=2) )

( dfp$kurt.xoc[index] <- e1071::kurtosis(xoc, type=2) )

( dfp$flor.xoc[index] <- table(xoc)[1] / length(xoc) )

( dfp$ceil.xoc[index] <- table(xoc)[length(table(xoc))] / length(xoc) )

( dfp$cor.sim1.xo1[index] <- cor(SIM1,xo1) )

( dfp$cor.sim2.xo2[index] <- cor(SIM2,xo2) )

### CREATE PERCEIVED CHANGE WITH PRESENT STATE BIAS

( mn.psb <- par.psb[k11] )

( sd.psb <- (0.5 - abs(0.5 - par.psb[k11]))/4 )

psb <- rnorm(N,mn.psb,sd.psb)

psb[psb<0] <- 0

psb[psb>1] <- 1

min(psb)

max(psb)

# Weighted theta change is composed of a proportion (psb) of theta T2 - mean T1

# and a proportion (1-psb) of theta change.

# The mean of theta T1 is subtracted from theta T2.

# This way of weighting ensures that the weighted change retains the same

# (theta) metric as theta T1 and theta T2

# However, weighting can impact the sample proportion improved

# Note: we assume that the TR item targets the trait underlying the SIM

tetch.wgt <- psb*(tet2s - mean(tet1s)) + (1-psb)*tetchs # weighted change

mean(tetchs)

sd(tetchs)

mean(tetch.wgt)

sd(tetch.wgt)

# Add ERROR to the weighted change: PERCEIVED CHANGE

( rel.trs <- par.rel.trs[k8] ) # reliability of the TRs

( sd.ch.error <- sqrt(((1-rel.trs)/rel.trs)*sd(tetch.wgt)^2) )

tetch.error <- rnorm(N, 0, sd.ch.error)

tetch.prc <- tetch.wgt + tetch.error

mean(tetch.wgt)

sd(tetch.wgt)

mean(tetch.prc)

sd(tetch.prc)

var(tetch.wgt)/var(tetch.prc) # reliability of the perceived change

cor(tetch.wgt,tetch.prc)^2 # reliability of the perceived change

# Create iMIC distribution

imic <- rnorm(N, par.mn.imic, par.sd.imic)

( dfp$mn.imic[index] <- mean(imic) )

( dfp$sd.imic[index] <- sd(imic) )

# create TRANSITION RATINGS -- DICHOTOMOUS

trs <- numeric(N)

trs[tetch.prc > imic] <- 1

table(trs)

# TR-change correlations

( dfp$cor.simdif.trs[index] <- cor(SIM.dif,trs) ) # point-biserial

( dfp$pcor.simdif.trs[index] <- psych::biserial(SIM.dif,trs) ) # biserial

# proportion truly improved based on true theta change

trs.tru <- numeric(N)

trs.tru[tetchs > imic] <- 1

table(trs.tru)

( dfp$prop.imp.tru[index] <- mean(trs.tru) )

# proportion improved based on perceived change

( dfp$prop.imp.trs[index] <- q <- mean(trs) )

( p <- log(q/(1-q)) ) # p = logodds(pre)

#########################

### Create different datasets

### dat.A, data with 1 random AUX item

# randomly select 1 item

( dfp$item.x[index] <- itemx <- sample(1:10,1) )

dat.A <- data.frame(SIM1,SIM2,trs,dat1[,itemx],dat2[,itemx])

names(dat.A)[4:5] <- c("v1", "v2")

head(dat.A)

### dat.B, model with AUX sum scores

dat.B <- data.frame(SIM1,SIM2,trs,xo1,xo2)

head(dat.B)

### dat.C, model with AUX sum scores, suitable for measurement invar. model

# Ensure that SIM variables have the same number of response options

# collapse response options if cells have less than "min.res" observations

table(SIM1)

length(table(SIM1))

table(SIM2)

length(table(SIM2))

# Ensure that the smallest SIM score is 1.

( minop <- min(min(SIM1),min(SIM2)) )

SIM1 <- SIM1 - minop+1

SIM2 <- SIM2 - minop+1

( minop <- min(min(SIM1),min(SIM2)) )

( maxop <- max(max(SIM1),max(SIM2)) )

( noptions <- maxop - minop + 1 )

( min.resp <- 3 )

mat <- matrix(rep(NA,noptions*2),ncol=2)

colnames(mat) <- c("T1","T2")

rownames(mat) <- rep(minop:maxop,1)

# mat

hlp <- data.frame(SIM1,SIM2)

for(j in 1:2) {

for(i in minop:maxop) {

mat[i,j] <- cnt <- length(hlp[,j][hlp[,j]==i])

if(cnt<min.resp & i==minop) {

SIM1[SIM1 <= i] <- i+1

SIM2[SIM2 <= i] <- i+1

}

if(cnt<min.resp & i==maxop) {

SIM1[SIM1 >= i] <- i-1

SIM2[SIM2 >= i] <- i-1

}

}

}

hlp <- data.frame(SIM1,SIM2)

for(j in 1:2) {

for(i in minop:maxop) {

mat[i,j] <- cnt <- length(hlp[,j][hlp[,j]==i])

if(cnt<min.resp & i==minop+1) {

SIM1[SIM1 <= i] <- i+1

SIM2[SIM2 <= i] <- i+1

}

if(cnt<min.resp & i==maxop-1) {

SIM1[SIM1 >= i] <- i-1

SIM2[SIM2 >= i] <- i-1

}

}

}

hlp <- data.frame(SIM1,SIM2)

for(j in 1:2) {

for(i in minop:maxop) {

mat[i,j] <- cnt <- length(hlp[,j][hlp[,j]==i])

if(cnt<min.resp & i==minop+2) {

SIM1[SIM1 <= i] <- i+1

SIM2[SIM2 <= i] <- i+1

}

if(cnt<min.resp & i==maxop-2) {

SIM1[SIM1 >= i] <- i-1

SIM2[SIM2 >= i] <- i-1

}

}

}

hlp <- data.frame(SIM1,SIM2)

for(j in 1:2) {

for(i in minop:maxop) {

mat[i,j] <- cnt <- length(hlp[,j][hlp[,j]==i])

if(cnt<min.resp & i==minop+3) {

SIM1[SIM1 <= i] <- i+1

SIM2[SIM2 <= i] <- i+1

}

if(cnt<min.resp & i==maxop-3) {

SIM1[SIM1 >= i] <- i-1

SIM2[SIM2 >= i] <- i-1

}

}

}

table(SIM1)

table(SIM2)

dat.C <- data.frame(SIM1,SIM2,trs,xo1,xo2)

head(dat.C)

### ANALYSES

length(table(SIM1))

if(length(table(SIM1))==10) {

model_2 <-

'SIM1 + SIM2 | b1*t1+b2*t2+b3*t3+b4*t4+b5*t5+b6*t6+b7*t7+b8*t8+b9*t9' }

if(length(table(SIM1))==9) {

model_2 <-

'SIM1 + SIM2 | b1*t1+b2*t2+b3*t3+b4*t4+b5*t5+b6*t6+b7*t7+b8*t8' }

if(length(table(SIM1))==8) {

model_2 <-

'SIM1 + SIM2 | b1*t1+b2*t2+b3*t3+b4*t4+b5*t5+b6*t6+b7*t7' }

if(length(table(SIM1))==7) {

model_2 <-

'SIM1 + SIM2 | b1*t1+b2*t2+b3*t3+b4*t4+b5*t5+b6*t6' }

if(length(table(SIM1))==6) {

model_2 <-

'SIM1 + SIM2 | b1*t1+b2*t2+b3*t3+b4*t4+b5*t5' }

if(length(table(SIM1))==5) {

model_2 <-

'SIM1 + SIM2 | b1*t1+b2*t2+b3*t3+b4*t4' }

model_2

### MIC predicted

mylogit <- glm(trs ~ SIM.dif, family = "binomial")

C <- coef(mylogit)[1] # intercept coefficient C

B <- coef(mylogit)[2] # regression coefficient B

( dfp$mic.pred[index] <- mic.pred <- (p-C)/B ) # MIC(predicted)

### MIC adjusted (2017)

( sf <- (0.09+0.103*cor(SIM.dif,trs)) * sd(SIM.dif) )

( dfp$mic.adj.old[index] <- mic.pred - sf * p )

### Model A.

model <- '

# factors

F1 =~ SIM1 + v1 + trs

F2 =~ SIM2 + v2 + trs

# Correlated errors over time

SIM1 ~~ SIM2

v1 ~~ v2

'

try( fit <- cfa(model, data=dat.A, ordered=c("v1","v2","trs")), silent=T )

if(exists("fit")==TRUE) {

if(fit@optim$converged==TRUE) {

if(lavInspect(fit, "post.check")==TRUE) {

# summary(fit)

( dfp$fit.cfi.ma[index] <- fitMeasures(fit, fit.measures="cfi.scaled") )

( dfp$fit.tli.ma[index] <- fitMeasures(fit, fit.measures="tli.scaled") )

( dfp$fit.rms.ma[index] <- fitMeasures(fit, fit.measures="rmsea.scaled") )

( dfp$fit.srmr.ma[index] <- fitMeasures(fit, fit.measures="srmr") )

pe <- parameterEstimates(fit, rsquare=T)

# pe

( dfp$rel.trs.ma[index] <- rel.trs <- pe$est[pe$lhs=="trs" & pe$op=="r2"] )

### Improved adjusted MIC (2022)

( rf <- (0.8/rel.trs - 0.5) * sd(SIM.dif) * cor(SIM.dif,trs) )

( dfp$mic.adj.ma[index] <- mic.pred - rf * p )

rm(pe)

rm(fit)

}}}

### Model B.

model <- '

# factors

F1 =~ SIM1 + xo1 + trs

F2 =~ SIM2 + xo2 + trs

# Correlated errors over time

SIM1 ~~ SIM2

xo1 ~~ xo2

'

try( fit <- cfa(model, data=dat.B, ordered="trs"), silent=T)

if(exists("fit")==TRUE) {

if(fit@optim$converged==TRUE) {

if(lavInspect(fit, "post.check")==TRUE) {

# summary(fit)

( dfp$fit.cfi.mb[index] <- fitMeasures(fit, fit.measures="cfi.scaled") )

( dfp$fit.tli.mb[index] <- fitMeasures(fit, fit.measures="tli.scaled") )

( dfp$fit.rms.mb[index] <- fitMeasures(fit, fit.measures="rmsea.scaled") )

( dfp$fit.srmr.mb[index] <- fitMeasures(fit, fit.measures="srmr") )

pe <- parameterEstimates(fit, rsquare=T)

# pe

( dfp$rel.trs.mb[index] <- rel.trs <- pe$est[pe$lhs=="trs" & pe$op=="r2"] )

## Improved adjusted MIC

rf <- (0.8/rel.trs - 0.5) * sd(SIM.dif) * dfp$cor.simdif.trs[index]

( dfp$mic.adj.mb[index] <- mic.pred - rf * p )

rm(pe)

rm(fit)

}}}

### Model C. Model with AUX sum scores, and measurement invariance

# data.frame(names(dat.C))

model_1 <- '

# factors

F1 =~ a1*SIM1 + xo1 + f1*trs

F2 =~ a1*SIM2 + xo2 + f2*trs

# Thresholds

# refer to model_2

# Correlated errors over time

SIM1 ~~ SIM2

xo1 ~~ xo2

# Variances/covariances

F1 ~~ 1*F1

F2 ~~ NA*F2 + var.F2*F2

F1 ~~ cov.F1F2*F2

SIM2 ~~ NA*SIM2

# Means/intercepts

F1 ~ 0*1

F2 ~ mn.ch*1

# Derived values

mn.change := mn.ch

sd.change := sqrt(1 + var.F2 - 2*cov.F1F2)

trs | thr.trs*t1

mic := thr.trs/f2

psb := f1/f2+1

'

model <- paste0(model_1,model_2)

try( fit <- cfa(model, data=dat.C, std.lv=T, ordered=c("SIM1","SIM2","trs"),

parameterization="theta"), silent=T)

if(exists("fit")==TRUE) {

if(fit@optim$converged==TRUE) {

if(lavInspect(fit, "post.check")==TRUE) {

# summary(fit)

( dfp$fit.cfi.mc[index] <- fitMeasures(fit, fit.measures="cfi.scaled") )

( dfp$fit.tli.mc[index] <- fitMeasures(fit, fit.measures="tli.scaled") )

( dfp$fit.rms.mc[index] <- fitMeasures(fit, fit.measures="rmsea.scaled") )

( dfp$fit.srmr.mc[index] <- fitMeasures(fit, fit.measures="srmr") )

pe <- parameterEstimates(fit, rsquare=T)

# pe

( dfp$mn.change.mc[index] <- pe$est[pe$label=="mn.change"] )

( dfp$sd.change.mc[index] <- pe$est[pe$label=="sd.change"] )

( dfp$rel.trs.mc[index] <- rel.trs <- pe$est[pe$lhs=="trs" & pe$op=="r2"] )

( dfp$psb.mc[index] <- pe$est[pe$label=="psb"] )

( dfp$mic.theta.mc[index] <- MIC.theta <- pe$est[pe$label=="mic"] )

## MIC based on SD of the true SIM score at T1

( dfp$rel.sim1.mc[index] <- rel.sim1 <- pe$est[pe$lhs=="SIM1" & pe$op=="r2"] )

# var(SIM1)*rel.sim1 # true variance of SIP1

# sqrt(var(SIM1)*rel.sip1) # SD of the SIM1 true score

( dfp$mic.lcfa.mc[index] <- sqrt(var(SIM1)*rel.sim1)*MIC.theta )

## Improved adjusted MIC

rf <- (0.8/rel.trs - 0.5) * sd(SIM.dif) * dfp$cor.simdif.trs[index]

( dfp$mic.adj.mc[index] <- mic.pred - rf * p )

rm(pe)

rm(fit)

}}}

}}}}}}}}}}}}

end.time <- Sys.time()

time.taken <- end.time - start.time

time.taken

beep(5)

#########################################################################

# write.table(dfp , file = "E:/Simulations_15552.txt",

# sep = " ", row.names = F, col.names = T)

# write.table(Randomseed , file = "E:/Randomseeds_15552.txt",

# sep = " ", row.names = F, col.names = T)

# 8. R-code for the MIC of single-item measures

# This R-code let you estimate the MIC for single-item measures (SIMs) using

# the APM-method and the LCFA-method (using LCFA-model C, see the article).

# Data preparation:

# Provide a text file with the following variables:

# - the SIM scores at baseline (T1); name the variable "sim1",

# - the SIM scores at follow-up (T2); name the variable "sim2",

# - the AUX scores at baseline (T1); name the variable "aux1",

# - the AUX scores at follow-up (T2); name the variable "aux2",

# - the transition ratings, dichotomized; name the variable "trs".

# The order of the variables is not important.

# If necessary, reverse the coding so that the scoring direction of all

# variables is: higher = better.

rm(list=ls(all=TRUE)) # clear work directory

library(lavaan)

library(DescTools)

# Read the data

datx <- read.table(file.choose(), header=T)

# Select complete cases if necessary

datx <- datx[complete.cases(datx),]

dim(datx) # see: rows (cases) and columns (variables)

data.frame(names(datx))

# Means and SD of the SIMs

mean(datx$sim1)

sd(datx$sim1)

mean(datx$sim2)

sd(datx$sim2)

### Calcualate MIC(pred) in the original metric of the SIM

sim.dif <- datx$sim2 - datx$sim1 # change score

trs <- datx$trs

( cor.simdif.trs <- cor(sim.dif, trs) ) # point-biserial correlation

# proportion improved

( q <- mean(trs) )

( p <- log(q/(1-q)) ) # p = logodds(pre)

mylogit <- glm(trs ~ sim.dif, family = "binomial")

C <- coef(mylogit)[1] # intercept coefficient C

B <- coef(mylogit)[2] # regression coefficient B

( mic.pred <- (p-C)/B ) # MIC(predicted)

# If the SIM is not an ordinal variable with 12 response categories max,

# apply linearly transformation (see the article).

# In this example division by 10 is used.

sim1 <- datx$sim1 / 10

sim2 <- datx$sim2 / 10

aux1 <- datx$aux1

aux2 <- datx$aux2

# Ensure that minimum option of SIM variables is 1,

# and that the SIM response categories are the same at T1 and T2.

( minop <- min(min(sim1),min(sim2)) )

sim1 <- sim1 - minop+1

sim2 <- sim2 - minop+1

( minop <- min(min(sim1),min(sim2)) )

( maxop <- max(max(sim1),max(sim2)) )

( noptions <- maxop - minop + 1 )

( min.resp <- 3 )

mat <- matrix(rep(NA,noptions*2),ncol=2)

colnames(mat) <- c("T1","T2")

rownames(mat) <- rep(minop:maxop,1)

# mat

hlp <- data.frame(sim1,sim2)

for(j in 1:2) {

for(i in 1:maxop) {

mat[i,j] <- cnt <- length(hlp[,j][hlp[,j]==i])

if(cnt<min.resp & i <= minop) {

sim1[sim1==i] <- i+1

sim2[sim2==i] <- i+1

}

if(cnt<min.resp & i >=maxop) {

sim1[sim1==i] <- i-1

sim2[sim2==i] <- i-1

}

}

}

hlp <- data.frame(sim1,sim2)

for(j in 1:2) {

for(i in 1:maxop) {

mat[i,j] <- cnt <- length(hlp[,j][hlp[,j]==i])

if(cnt<min.resp & i <= minop+1) {

sim1[sim1==i] <- i+1

sim2[sim2==i] <- i+1

}

if(cnt<min.resp & i >= maxop-1) {

sim1[sim1==i] <- i-1

sim2[sim2==i] <- i-1

}

}

}

hlp <- data.frame(sim1,sim2)

for(j in 1:2) {

for(i in 1:maxop) {

mat[i,j] <- cnt <- length(hlp[,j][hlp[,j]==i])

if(cnt<min.resp & i <= minop+2) {

sim1[sim1==i] <- i+1

sim2[sim2==i] <- i+1

}

if(cnt<min.resp & i >= maxop-2) {

sim1[sim1==i] <- i-1

sim2[sim2==i] <- i-1

}

}

}

hlp <- data.frame(sim1,sim2)

for(j in 1:2) {

for(i in 1:maxop) {

mat[i,j] <- cnt <- length(hlp[,j][hlp[,j]==i])

if(cnt<min.resp & i <= minop+3) {

sim1[sim1==i] <- i+1

sim2[sim2==i] <- i+1

}

if(cnt<min.resp & i >= maxop-3) {

sim1[sim1==i] <- i-1

sim2[sim2==i] <- i-1

}

}

}

# Check of sim1 and sim2 have the same response categories.

table(sim1)

table(sim2)

# Create a new data file with the transformed variables

dat <- data.frame(sim1,sim2,aux1,aux2,trs)

# Create the LCFA model for lavaan

length(table(sim1))

if(length(table(sim1))==12) {

model_2 <-

'sim1 + sim2 | b1*t1+b2*t2+b3*t3+b4*t4+b5*t5+b6*t6+b7*t7+b8*t8+b9*t9+b10*t10+b11*t11+b12*t12' }

if(length(table(sim1))==11) {

model_2 <-

'sim1 + sim2 | b1*t1+b2*t2+b3*t3+b4*t4+b5*t5+b6*t6+b7*t7+b8*t8+b9*t9+b10*t10+b11*t11' }

if(length(table(sim1))==10) {

model_2 <-

'sim1 + sim2 | b1*t1+b2*t2+b3*t3+b4*t4+b5*t5+b6*t6+b7*t7+b8*t8+b9*t9+b10*t10' }

if(length(table(sim1))==10) {

model_2 <-

'sim1 + sim2 | b1*t1+b2*t2+b3*t3+b4*t4+b5*t5+b6*t6+b7*t7+b8*t8+b9*t9' }

if(length(table(sim1))==9) {

model_2 <-

'sim1 + sim2 | b1*t1+b2*t2+b3*t3+b4*t4+b5*t5+b6*t6+b7*t7+b8*t8' }

if(length(table(sim1))==8) {

model_2 <-

'sim1 + sim2 | b1*t1+b2*t2+b3*t3+b4*t4+b5*t5+b6*t6+b7*t7' }

if(length(table(sim1))==7) {

model_2 <-

'sim1 + sim2 | b1*t1+b2*t2+b3*t3+b4*t4+b5*t5+b6*t6' }

if(length(table(sim1))==6) {

model_2 <-

'sim1 + sim2 | b1*t1+b2*t2+b3*t3+b4*t4+b5*t5' }

if(length(table(sim1))==5) {

model_2 <-

'sim1 + sim2 | b1*t1+b2*t2+b3*t3+b4*t4' }

model_2

model_1 <- '

# factors

F1 =~ a1*sim1 + aux1 + f1*trs

F2 =~ a1*sim2 + aux2 + f2*trs

# Thresholds

# refer to model_2

# Correlated errors over time

sim1 ~~ sim2

aux1 ~~ aux2

# Variances/covariances

F1 ~~ 1*F1

F2 ~~ NA*F2 + var.F2*F2

F1 ~~ cov.F1F2*F2

sim2 ~~ NA*sim2

# Means/intercepts

F1 ~ 0*1

F2 ~ mn.ch*1

# Derived values

mn.change := mn.ch

sd.change := sqrt(1 + var.F2 - 2*cov.F1F2)

trs | thr.trs*t1

mic := thr.trs/f2

psb := f1/f2+1

'

model <- paste0(model_1,model_2)

# Fit the LCFA-model

fit <- cfa(model, data=dat, std.lv=T, ordered=c("sim1","sim2","trs"),

parameterization="theta")

# Observe the fit indices

fitMeasures(fit, fit.measures="cfi.scaled")

fitMeasures(fit, fit.measures="tli.scaled")

fitMeasures(fit, fit.measures="rmsea.scaled")

fitMeasures(fit, fit.measures="srmr")

# Extract the estimates of interest

pe <- parameterEstimates(fit, rsquare=T)

# mean latent change

pe$est[pe$label=="mn.change"]

# SD of the latent change

pe$est[pe$label=="sd.change"]

# TRs reliability

( rel.trs <- pe$est[pe$lhs=="trs" & pe$op=="r2"] )

# PSB (present state bias)

pe$est[pe$label=="psb"]

# latent MIC

( MIC.theta <- pe$est[pe$label=="mic"] )

### Calculate the improved adjusted MIC (in the original metric of the SIMs)

rf <- (0.8/rel.trs - 0.5) * sd(sim.dif) * cor.simdif.trs

( mic.adj <- mic.pred - rf * p )

### Calculate the LCFA-based MIC

# Reliability of SIM_T1

( rel.sim1 <- pe$est[pe$lhs=="sim1" & pe$op=="r2"] )

# Calculate the MIC in the transformed metric of SIM

( mic.tf <- sqrt(var(sim1)*rel.sim1)*MIC.theta )

# Back-transform MIC to the metric of the original SIM scale

( mic.tf * 10 )

############ START BOOTSTRAP LOOP #############

set.seed(123)

nb = 2000 # number of bootstrap samples

mn.sim1 <- as.numeric(rep(NA, nb))

mn.sim2 <- as.numeric(rep(NA, nb))

sd.sim1 <- as.numeric(rep(NA, nb))

sd.sim2 <- as.numeric(rep(NA, nb))

boot <- data.frame(mn.sim1,sd.sim1,mn.sim2,sd.sim2)

boot$sim1.aux1.cor <- as.numeric(rep(NA, nb))

boot$sim2.aux2.cor <- as.numeric(rep(NA, nb))

boot$prop.imp <- as.numeric(rep(NA, nb))

boot$cor.simdif.trs <- as.numeric(rep(NA, nb))

boot$mic.pred <- as.numeric(rep(NA, nb))

boot$cfi <- as.numeric(rep(NA, nb))

boot$tli <- as.numeric(rep(NA, nb))

boot$rmsea <- as.numeric(rep(NA, nb))

boot$srmr <- as.numeric(rep(NA, nb))

boot$mn.lat.change <- as.numeric(rep(NA, nb))

boot$sd.lat.change <- as.numeric(rep(NA, nb))

boot$rel.trs <- as.numeric(rep(NA, nb))

boot$psb <- as.numeric(rep(NA, nb))

boot$mic.theta <- as.numeric(rep(NA, nb))

boot$mic.adj <- as.numeric(rep(NA, nb))

boot$rel.sim1 <- as.numeric(rep(NA, nb))

boot$mic.lcfa <- as.numeric(rep(NA, nb))

############

for(bt in 1:nb) {

print(bt)

# Create a new bootstrap sample

selection <- sample(1:nrow(datx), nrow(datx), replace=TRUE)

dat <- datx[selection,]

# Prevent continuation when proportion improved is <0.05 or >0.95

for(k in 1:10) {

if(mean(dat$trs)<0.05|mean(dat$trs)>0.95) {

selection <- sample(1:nrow(datx), nrow(datx), replace=TRUE)

dat <- datx[selection,]

}}

( boot$mn.sim1[bt] <- mean(dat$sim1) )

( boot$sd.sim1[bt] <- sd(dat$sim1) )

( boot$mn.sim2[bt] <- mean(dat$sim2) )

( boot$sd.sim2[bt] <- sd(dat$sim2) )

# SIM-AUX-Correlations

( boot$sim1.aux1.cor[bt] <- cor(dat$sim1,aux1) )

( boot$sim2.aux2.cor[bt] <- cor(dat$sim2,aux2) )

### Calcualate MIC(pred)

sim.dif <- dat$sim2 - dat$sim1 # change score

trs <- dat$trs

( boot$cor.simdif.trs[bt] <- COR <- cor(sim.dif, trs) ) # point-biserial correlation

# proportion improved

( boot$prop.imp[bt] <- q <- mean(trs) )

( p <- log(q/(1-q)) ) # p = logodds(pre)

mylogit <- glm(trs ~ sim.dif, family = "binomial")

C <- coef(mylogit)[1] # intercept coefficient C

B <- coef(mylogit)[2] # regression coefficient B

( boot$mic.pred[bt] <- mic.pred <- (p-C)/B ) # MIC(predicted)

# APPLY THE TRANSFORMATION IF NECESSARY

# Linearly transform SIM into ordinal 0-10 score

sim1 <- dat$sim1 / 10

sim2 <- dat$sim2 / 10

aux1 <- dat$aux1

aux2 <- dat$aux2

# Ensure that minimal option of SIM variables is 1

( minop <- min(min(sim1),min(sim2)) )

sim1 <- sim1 - minop+1

sim2 <- sim2 - minop+1

( minop <- min(min(sim1),min(sim2)) )

( maxop <- max(max(sim1),max(sim2)) )

( noptions <- maxop - minop + 1 )

( min.resp <- 3 )

mat <- matrix(rep(NA,noptions*2),ncol=2)

colnames(mat) <- c("T1","T2")

rownames(mat) <- rep(minop:maxop,1)

# mat

hlp <- data.frame(sim1,sim2)

for(j in 1:2) {

for(i in 1:maxop) {

mat[i,j] <- cnt <- length(hlp[,j][hlp[,j]==i])

if(cnt<min.resp & i==minop) {

sim1[sim1 <= i] <- i+1

sim2[sim2 <= i] <- i+1

}

if(cnt<min.resp & i==maxop) {

sim1[sim1 >= i] <- i-1

sim2[sim2 >= i] <- i-1

}

}

}

hlp <- data.frame(sim1,sim2)

for(j in 1:2) {

for(i in 1:maxop) {

mat[i,j] <- cnt <- length(hlp[,j][hlp[,j]==i])

if(cnt<min.resp & i==minop+1) {

sim1[sim1 <= i] <- i+1

sim2[sim2 <= i] <- i+1

}

if(cnt<min.resp & i==maxop-1) {

sim1[sim1 >= i] <- i-1

sim2[sim2 >= i] <- i-1

}

}

}

hlp <- data.frame(sim1,sim2)

for(j in 1:2) {

for(i in 1:maxop) {

mat[i,j] <- cnt <- length(hlp[,j][hlp[,j]==i])

if(cnt<min.resp & i==minop+2) {

sim1[sim1 <= i] <- i+1

sim2[sim2 <= i] <- i+1

}

if(cnt<min.resp & i==maxop-2) {

sim1[sim1 >= i] <- i-1

sim2[sim2 >= i] <- i-1

}

}

}

hlp <- data.frame(sim1,sim2)

for(j in 1:2) {

for(i in 1:maxop) {

mat[i,j] <- cnt <- length(hlp[,j][hlp[,j]==i])

if(cnt<min.resp & i <= minop+3) {

sim1[sim1==i] <- i+1

sim2[sim2==i] <- i+1

}

if(cnt<min.resp & i >= maxop-3) {

sim1[sim1==i] <- i-1

sim2[sim2==i] <- i-1

}

}

}

table(sim1)

table(sim2)

# Re-create "dat" with the transformed variables

dat <- data.frame(sim1,sim2,aux1,aux2,trs)

length(table(sim1))

if(length(table(sim1))==12) {

model_2 <-

'sim1 + sim2 | b1*t1+b2*t2+b3*t3+b4*t4+b5*t5+b6*t6+b7*t7+b8*t8+b9*t9+b10*t10+b11*t11+b12*t12' }

if(length(table(sim1))==11) {

model_2 <-

'sim1 + sim2 | b1*t1+b2*t2+b3*t3+b4*t4+b5*t5+b6*t6+b7*t7+b8*t8+b9*t9+b10*t10+b11*t11' }

if(length(table(sim1))==10) {

model_2 <-

'sim1 + sim2 | b1*t1+b2*t2+b3*t3+b4*t4+b5*t5+b6*t6+b7*t7+b8*t8+b9*t9+b10*t10' }

if(length(table(sim1))==10) {

model_2 <-

'sim1 + sim2 | b1*t1+b2*t2+b3*t3+b4*t4+b5*t5+b6*t6+b7*t7+b8*t8+b9*t9' }

if(length(table(sim1))==9) {

model_2 <-

'sim1 + sim2 | b1*t1+b2*t2+b3*t3+b4*t4+b5*t5+b6*t6+b7*t7+b8*t8' }

if(length(table(sim1))==8) {

model_2 <-

'sim1 + sim2 | b1*t1+b2*t2+b3*t3+b4*t4+b5*t5+b6*t6+b7*t7' }

if(length(table(sim1))==7) {

model_2 <-

'sim1 + sim2 | b1*t1+b2*t2+b3*t3+b4*t4+b5*t5+b6*t6' }

if(length(table(sim1))==6) {

model_2 <-

'sim1 + sim2 | b1*t1+b2*t2+b3*t3+b4*t4+b5*t5' }

if(length(table(sim1))==5) {

model_2 <-

'sim1 + sim2 | b1*t1+b2*t2+b3*t3+b4*t4' }

model_2

model_1 <- '

# factors

F1 =~ a1*sim1 + aux1 + f1*trs

F2 =~ a1*sim2 + aux2 + f2*trs

# Thresholds

# refer to model_2

# Correlated errors over time

sim1 ~~ sim2

aux1 ~~ aux2

# Variances/covariances

F1 ~~ 1*F1

F2 ~~ NA*F2 + var.F2*F2

F1 ~~ cov.F1F2*F2

sim2 ~~ NA*sim2

# Means/intercepts

F1 ~ 0*1

F2 ~ mn.ch*1

# Derived values

mn.change := mn.ch

sd.change := sqrt(1 + var.F2 - 2*cov.F1F2)

trs | thr.trs*t1

mic := thr.trs/f2

psb := f1/f2+1

'

model <- paste0(model_1,model_2)

try(fit <- cfa(model, data=dat, std.lv=T, ordered=c("sim1","sim2","trs"),

parameterization="theta"), silent=T)

if(exists("fit")==TRUE) {

if(fit@optim$converged==TRUE) {

if(lavInspect(fit, "post.check")==TRUE) {

summary(fit)

( boot$cfi[bt] <- fitMeasures(fit, fit.measures="cfi.scaled") )

( boot$tli[bt] <- fitMeasures(fit, fit.measures="tli.scaled") )

( boot$rmsea[bt] <- fitMeasures(fit, fit.measures="rmsea.scaled") )

( boot$srmr[bt] <- fitMeasures(fit, fit.measures="srmr") )

pe <- parameterEstimates(fit, rsquare=T)

( boot$mn.lat.change[bt] <- pe$est[pe$label=="mn.change"] )

( boot$sd.lat.change[bt] <- pe$est[pe$label=="sd.change"] )

( boot$rel.trs[bt] <- rel.trs <- pe$est[pe$lhs=="trs" & pe$op=="r2"] )

( boot$psb[bt] <- pe$est[pe$label=="psb"] )

( boot$mic.theta[bt] <- MIC.theta <- pe$est[pe$label=="mic"] )

# Improved adjusted MIC

rf <- (0.8/rel.trs - 0.5) * sd(sim.dif) * COR

( boot$mic.adj[bt] <- mic.pred - rf * p )

# MIC LCFA

( boot$rel.sim1[bt] <- rel.sim1 <- pe$est[pe$lhs=="sim1" & pe$op=="r2"] )

( mic.tf <- sqrt(var(sim1)*rel.sim1)*MIC.theta )

# Back-transform MIC to original SIM scale

( boot$mic.lcfa[bt] <- mic.tf * 10 )

rm(pe)

rm(fit)

}}}

}

############ END BOOTSTRAP LOOP #############

write.table(boot , file = "D:/Boot-results.txt",

sep = " ", row.names = F, col.names = T)

summary(boot)

data.frame(names(boot))

round(mean(boot$mn.sim1, na.rm=T),1)

round(quantile(boot$mn.sim1, c(0.025,0.975), na.rm=T),1)

round(mean(boot$sd.sim1, na.rm=T),1)

round(quantile(boot$sd.sim1, c(0.025,0.975), na.rm=T),1)

round(mean(boot$mn.sim2, na.rm=T),1)

round(quantile(boot$mn.sim2, c(0.025,0.975), na.rm=T),1)

round(mean(boot$sd.sim2, na.rm=T),1)

round(quantile(boot$sd.sim2, c(0.025,0.975), na.rm=T),1)

round(mean(boot$sim1.aux1.cor, na.rm=T),2)

round(quantile(boot$sim1.aux1.cor, c(0.025,0.975), na.rm=T),2)

round(mean(boot$sim2.aux2.cor, na.rm=T),2)

round(quantile(boot$sim2.aux2.cor, c(0.025,0.975), na.rm=T),2)

round(mean(boot$prop.imp, na.rm=T),2)

round(quantile(boot$prop.imp, c(0.025,0.975), na.rm=T),2)

round(mean(boot$cor.simdif.trs, na.rm=T),2)

round(quantile(boot$cor.simdif.trs, c(0.025,0.975), na.rm=T),2)

round(mean(boot$mic.pred, na.rm=T),1)

round(quantile(boot$mic.pred, c(0.025,0.975), na.rm=T),1)

round(mean(boot$cfi, na.rm=T),3)

round(quantile(boot$cfi, c(0.025,0.975), na.rm=T),3)

round(mean(boot$tli, na.rm=T),3)

round(quantile(boot$tli, c(0.025,0.975), na.rm=T),3)

round(mean(boot$rmsea, na.rm=T),3)

round(quantile(boot$rmsea, c(0.025,0.975), na.rm=T),3)

round(mean(boot$srmr, na.rm=T),3)

round(quantile(boot$srmr, c(0.025,0.975), na.rm=T),3)

round(mean(boot$mn.lat.change, na.rm=T),2)

round(quantile(boot$mn.lat.change, c(0.025,0.975), na.rm=T),2)

round(mean(boot$sd.lat.change, na.rm=T),2)

round(quantile(boot$sd.lat.change, c(0.025,0.975), na.rm=T),2)

round(mean(boot$rel.trs, na.rm=T),3)

round(quantile(boot$rel.trs, c(0.025,0.975), na.rm=T),3)

round(mean(boot$psb, na.rm=T),3)

round(quantile(boot$psb, c(0.025,0.975), na.rm=T),3)

round(mean(boot$mic.theta, na.rm=T),2)

round(quantile(boot$mic.theta, c(0.025,0.975), na.rm=T),2)

round(mean(boot$mic.adj, na.rm=T),1)

round(quantile(boot$mic.adj, c(0.025,0.975), na.rm=T),1)

round(mean(boot$rel.sim1, na.rm=T),3)

round(quantile(boot$rel.sim1, c(0.025,0.975), na.rm=T),3)

round(mean(boot$mic.lcfa, na.rm=T),1)

round(quantile(boot$mic.lcfa, c(0.025,0.975), na.rm=T),1)

1. Guyatt et al., doi: 10.1016/s0895-4356(02)00435-3 [↑](#footnote-ref-1)
2. Terluin et al., doi: 10.1016/j.jclinepi.2021.12.024 [↑](#footnote-ref-2)
3. Terluin et al., doi: 10.1007/s11136-024-03763-4 [↑](#footnote-ref-3)
4. Taken from: Terluin B, Trigg A, Fromy P, Schuller W, Terwee CB, Bjorner JB. Estimating anchor-based minimal important change using longitudinal confirmatory factor analysis. Qual Life Res 2024; 33(4): 963-973, Supplement, Sect. 1 [↑](#footnote-ref-4)
5. Terluin et al., doi: 10.1007/s11136-024-03763-4, Online Supplement, Fig. S4 [↑](#footnote-ref-5)
6. Terluin et al., doi: 10.1007/s11136-024-03763-4, Online Supplement, Fig. S4 [↑](#footnote-ref-6)
7. Terluin et al., doi: 10.1007/s11136-024-03763-4, Online Supplement, Fig. S4 [↑](#footnote-ref-7)
8. doi: 10.1016/j.jclinepi.2016.12.015 [↑](#footnote-ref-8)
9. doi: 10.1016/j.jclinepi.2022.04.018 [↑](#footnote-ref-9)
10. Note that, due to the link we simulated between the metric of the latent construct and the metric of the SIM (see Sect. 1), the true MIC-values (0.5 and 1.0) applied to both metrics. [↑](#footnote-ref-10)
11. e.g., doi: 10.1016/j.jclinepi.2007.03.012 [↑](#footnote-ref-11)
